# Iron dyshomeostasis and ferroptosis in Alzheimer’s disease: Molecular mechanisms of cell death and novel therapeutic drugs and targets for AD

**DOI:** 10.3389/fphar.2022.983623

**Published:** 2022-09-16

**Authors:** Yuan Zhang, Man Wang, Wenguang Chang

**Affiliations:** Institute for Translational Medicine, The Affiliated Hospital of Qingdao University, Qingdao University, Qingdao, China

**Keywords:** Alzheimer’s disease, ferroptosis, iron dyshomeostasis, Nrf2, signal pathways

## Abstract

Alzheimer’s disease (AD) is a degenerative disease of the central nervous system that is the most common type of senile dementia. Ferroptosis is a new type of iron-dependent programmed cell death identified in recent years that is different from other cell death forms. Ferroptosis is induced by excessive accumulation of lipid peroxides and reactive oxygen species (ROS) in cells. In recent years, it has been found that ferroptosis plays an important role in the pathological process of AD. Iron dyshomeostasis contribute to senile plaques (SP) deposition and neurofibrillary tangles (NFTs). Iron metabolism imbalance in brain and the dysfunction of endogenous antioxidant systems including system Xc- and glutathione peroxidase (GPX) are closely related to the etiopathogenesis of AD. Dysfunction of nuclear receptor coactivator 4 (NCOA4)-mediated ferritinophagy induced ferroptosis can accelerates the pathological process of AD. In addition, NRF2, through regulating the expression of a considerable number of genes related to ferroptosis, including genes related to iron and glutathione metabolism, plays an important role in the development of AD. Here, we review the potential interaction between AD and ferroptosis and the major pathways regulating ferroptosis in AD. We also review the active natural and synthetic compounds such as iron chelators, lipid peroxidation inhibitors and antioxidants available to treat AD by alleviating iron dyshomeostasis and preventing ferroptosis in mice and cell models to provide valuable information for the future treatment and prevention of AD.

## Introduction

Iron plays a fundamental role in maintaining several biological functions in the brain. In the brain, iron is essential for meeting the high metabolic and energy demands of neuronal tissue and is also involved in myelin synthesis, neurotransmitter synthesis and metabolism (Gerlach et al., 1994; Belaidi and Bush, 2016). Iron dyshomeostasis within the brain can cause oxidative stress and inflammatory responses, causing neurotoxicity and cell damage and ultimately neurological diseases (Ward et al., 2014).

Ferroptosis is a new type of programmed cell death identified in recent years that is different from other cell death forms, such as apoptosis, necrosis, autophagy or pyroptosis. Ferroptosis is induced by the iron-dependent accumulation of lipid peroxides, and reactive oxygen species (ROS) production, the depletion of glutathione (GSH), and inactivation of glutathione peroxidase 4 (GPX4) in cells (Dixon et al., 2012; Chen et al., 2021a).

Accumulating evidence indicates that Alzheimer’s disease (AD) pathology is closely related to iron dyshomeostasis and ferroptosis. Studies show that brain iron accumulation is more serious in special brain areas of patients with neurodegenerative diseases and induces oxidative stress and neuronal death. Iron metabolism imbalance in the brain and dysfunction of endogenous antioxidant systems, including GPX, are closely related to the etiopathogenesis of AD (Hambright et al., 2017). Patients with mild cognitive impairment (MCI) and a high Aβ plaque load exhibit higher cortical iron accumulation, which increases the risk of AD (van Bergen et al., 2016). Evidence of elevated iron and the production of lipid peroxidation in the AD brain indicates the important role of ferroptosis in the pathogenesis of AD. The features of ferroptosis can be detected in the brain tissues of AD patients and mouse models, such as abnormal iron accumulation, glutamate excitotoxicity and lipid peroxidation accumulation (Lyras et al., 1997; Huang et al., 2017; Ayton et al., 2021). Evidence has shown that ferritin levels in cerebrospinal fluid (CSF) are negatively associated with MCI and AD patients. Ferritin was strongly associated with CSF apolipoprotein E levels and was elevated by the Alzheimer’s risk allele APOE-ɛ4, indicating that iron imbalance can be one of the risk factors for AD. Iron dyshomeostasis and ferroptosis may play the important roles in neuronal death in AD (Ayton et al., 2015).

## Characteristics of ferroptosis in AD

The classic features of ferroptosis include the following: 1) specific changes in cellular morphology including a ferroptosis-triggered decrease in mitochondrial size, an increase in membrane density and a decrease in cristae number without obvious morphological changes in the nucleus and 2) specific changes in cellular composition including iron-promoted increases in lipid peroxides and ROS levels, depletion of GSH, inactivation of GPX4, and changes in the expression of several regulated genes (Dixon et al., 2012; Chen et al., 2021a).

### Iron metabolism is involved in ferroptosis in neuron cells

The balance of iron metabolism depends on the synergistic action of various iron metabolism-related proteins, and iron dyshomeostasis may lead to ferroptosis. The transferrin (Tf)/transferrin receptor (TfR) pathway is mainly responsible for the absorption of iron into cells. When the iron transporter function of the Tf/TfR pathway is abnormal, it will lead to oxidative stress and ferroptosis. Abnormal Tf and TfR1 expression can lead to neurodegenerative disorders (Senyilmaz et al., 2015). Studies have shown that overexpression of divalent metal ion transporter 1 (DMT1), as an iron-absorbing protein, leads to the iron deposition in substantia nigra and death of dopaminergic neurons (Zhang et al., 2017). Ferritin is the main intracellular iron storage protein composed of light chain protein and heavy chain protein, which stores iron in a redox inactive form and protects cells and tissues from oxidative damage. Iron dyshomeostasis caused by abnormal ferritin will induce ferroptosis (Park and Chung, 2019). Upon ferroptosis, ferritin in the cytoplasm undergoes ferritinophagy in the lysosome to further expand labile iron pool (LIP), and ferritinophagy enhances cysteine deficiency-induced ferroptosis. While total intracellular iron content may not change, LIP expansion predisposes cells to ferroptosis. Imbalances in iron metabolism lead to iron overload, which in turn generates free radicals through the Fenton and Haber-Weiss reactions, thereby activating ferroptosis (Kajarabille and Latunde-Dada, 2019).

### GSH/GPX4 is involved in ferroptosis by mediating lipid peroxidation in neuron cells

Massive accumulation of lipid peroxides to lethal levels is a necessary signal for ferroptosis, and the levels of lipid peroxide biomarkers are also elevated in neuronal cells in AD. Due to the accumulation of iron, the Fenton and Haber-Weiss reaction occurs in cells, and a large amount of ROS is generated, leading to lipid peroxidation (Kajarabille and Latunde-Dada, 2019). The enzyme GSH plays a key role in protecting cells from oxidative damage, and GSH-dependent GPX4 activity is important for regulating neuronal ferroptosis. GPX4 utilizes the thiol groups of GSH as electron donors and affects the antioxidant response of neuron cells. Therefore, GPX4 inactivation caused by GSH depletion increases intracellular lipid peroxidation and induces ferroptosis (Imai et al., 2017). Studies have shown that neuron-specific knockout of GPX4 leads to lipid peroxidation and the accumulation of intracellular ROS, eventually leading to neuronal death, suggesting that GPX4 may be a key regulator of ferroptosis in neuron cells (Hambright et al., 2017).

In the current study, the occurrence of neuronal ferroptosis was determined by evaluating morphological changes or pharmacological and molecular characteristics. However, changes in the levels of intracellular iron metabolism-related proteins and lipid peroxidation factors including downregulation of GSH and GPX4 and upregulation of lipid peroxidation markers such as MDA, isoprostanes, 4-HNE, malondialdehyde and acrolein are key markers of neuronal ferroptosis (Gegotek and Skrzydlewska, 2019).

The involvement of novel ferroptosis-related genes in AD was revealed by advanced biological sequencing methods. For example, Wang et al. found five hub genes (JUN, SLC2A1, TFRC, ALB, and NFE2L2) that are closely associated with ferroptosis in AD and can differentiate AD patients from controls; these genes can be used as potential ferroptosis-related biomarkers for disease diagnosis and therapeutic monitoring (Wang et al., 2022).

## Mechanisms of iron dyshomeostasis and ferroptosis leading to AD

### Iron dyshomeostasis contribute to senile plaques deposition and neurofibrillary tangles

Iron is an endogenous metal ion involved in many important physiological processes in the brain. Under physiological conditions, iron ions are absorbed, transported, transformed and excreted by the human body to maintain their normal levels. Once free iron ions are enriched, cells will produce excess ROS, leading to ferroptosis. Therefore, the increase in iron ion content may be one of the critical factors for AD pathogenesis. Studies have shown that abnormal iron metabolism is related to the pathogenesis of AD. Compared with controls, AD patients had elevated iron levels in the hippocampus, cortex, and basal ganglia regions of the brain and lower levels of iron in the cerebral cortex, brainstem, and cerebellum (Ramos et al., 2014). The iron content and ferritin protein expression in brain tissue were related to the degree of amyloid deposition, due to the level of APP, like ferritin, can be regulated by iron content at the translational level. Thus, the increased levels of iron content in the brain might affect the progression of AD (Rogers et al., 2008; Chen et al., 2019; Svobodova et al., 2019). A study by Rogers et al. showed that AD patients receiving intramuscular injection of the iron chelator deferoxamine (DFO) had a lesser degree of decline in activities of daily living than AD patients receiving placebo, indicating that abnormal iron metabolism can affect the progression of AD (Crapper McLachlan et al., 1991).

Accumulating evidence suggests that iron dyshomeostasis contribute to the aggregation of beta-amyloid (Aβ)-induced SP deposition and hyperphosphorylated tau proteins that form NFTs in the brain. SP and NFTs cover redox-active transition metals.

### Iron dyshomeostasis induces Aβ level elevation

**FIGURE 1:**
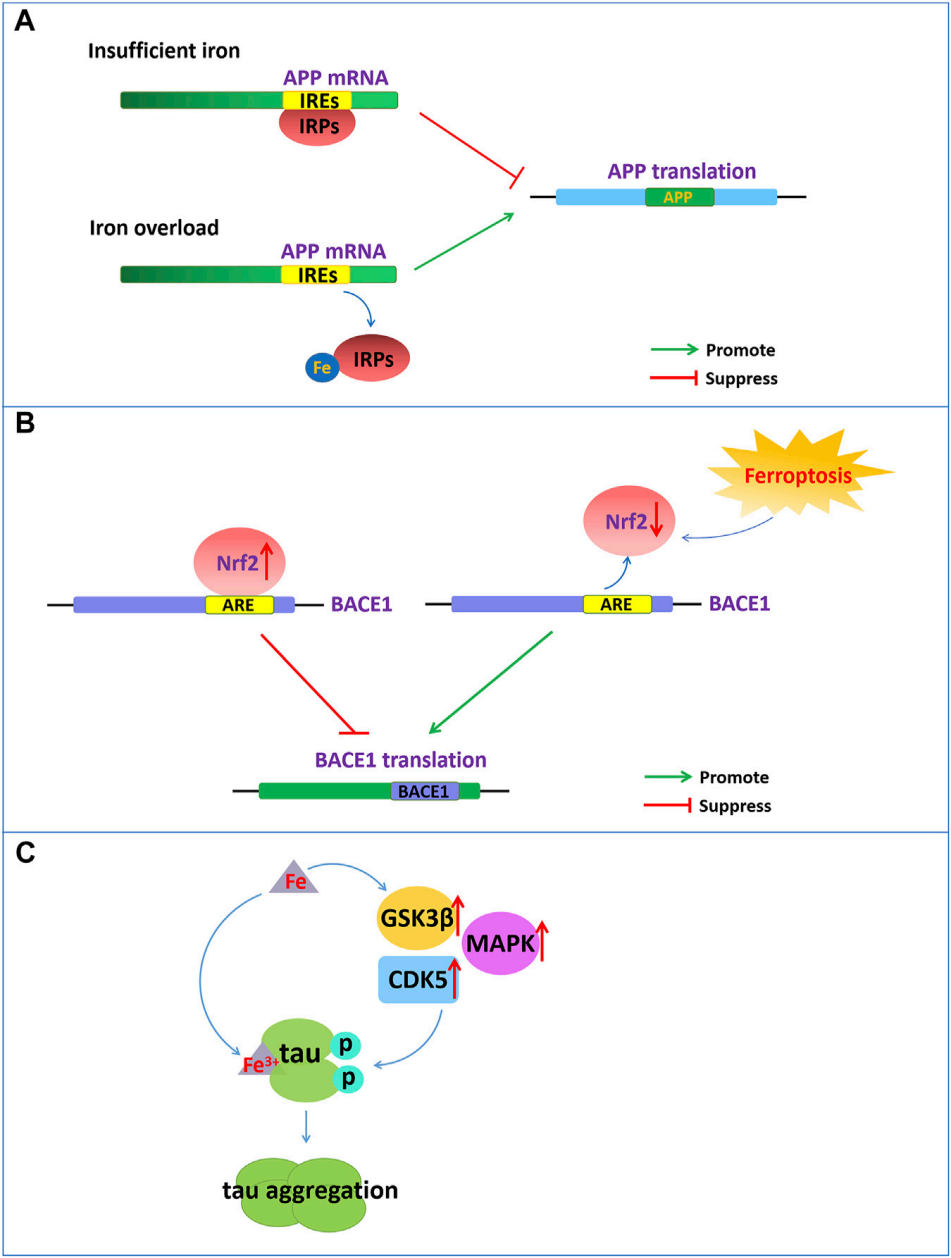
Iron dyshomeostasis contribute to senile plaques deposition and neurofibrillary tangles. **(A)** The 5′-UTR of APP mRNA contains IREs. APP can be posttranscriptionally regulated by IRP-IRE. Under conditions of insufficient iron, IRPs bind to IREs of APP mRNA and repress APP translation. During iron overload, iron can bind to IRPs, leading to the dissociation of IRPs from IREs and promoting the translation of APP. **(B)** NRF2, as a negative regulator of BACE1, represses the expression of BACE1 by binding to AREs. Nrf2 activation repress BACE1 transcription. In turn, NRF2 inactivation promote BACE1 transcription. **(C)** Increased cellular iron levels promote the activity of enzymes such as GSK3β, CDK5, and MAPK, which promote tau hyperphosphorylation. Fe3+ can promote the formation of tau fibrils by binding to the phosphorylated tau.

Aβ formation results from the amyloidogenic cleavage of human amyloid precursor protein (APP). APP is processed by two different pathways: the *a*- and γ-secretase-mediated nonamyloid protein production pathway and the β- and γ-secretase-mediated amyloid protein production pathway (O'Brien and Wong, 2011). APP is a type 1 transmembrane glycoprotein and a key precursor for the production of Aβ. APP can be sequentially cleaved by *a*-secretase or β-secretase first and then by the γ-secretase complex. Under normal physiological conditions, APP can be cleaved by *a*-secretase within the Aβ domain to release soluble APPα and preclude Aβ generation. However, once APP is cleaved first by β-secretase and then by the γ-secretase complex, it will eventually produce neurotoxic amyloid of 40–42 amino acids. The 5′-untranslated region (5′-UTR mRNA) of APP mRNA contains iron-responsive elements (IREs), which is regulated by intracellular iron content (Figure 1A). APP can be posttranscriptionally regulated by iron‐responsive‐element‐binding proteins (IRPs)-IREs. Under conditions of insufficient iron, IRPs bind tightly to IREs at the 5′-UTR site of APP mRNA and repress APP translation. During intracellular iron overload, iron can bind to IRPs, leading to the dissociation of IRPs from IREs and promoting the translation of APP, thereby increasing the level of APP protein and possibly producing excess Aβ. However, in turn, APP can interact with and stabilize ferroportin (Fpn) to facilitate iron export and maintain the homeostasis of iron concentration (Belaidi et al., 2018).

Iron also affects *a*-secretase activity and suppresses the nonamyloidogenic pathway, thereby increasing Aβ production through the amyloidogenic pathway. Furin plays a critical role in mediating APP processing by regulating the activation of *a*-secretase. Furin is a proprotein convertase enzyme that converts various proproteins to mature proteins, which are affected by iron content. Wichaiyo et al. demonstrated that iron affected the expression of furin by posttranslational regulation. In the iron overload condition, furin mRNA showed a significant reduction (Wichaiyo et al., 2015). Furin is an important regulator of *a*-secretase-associated APP processing (Hwang et al., 2006). Hwang et al. demonstrated that the levels of furin in the brains of AD patients and Tg2576 mice were significantly reduced, yet injection of furin-adenovirus into Tg2576 mouse brains markedly increased *a*-secretase activity and reduced Aβ production in infected brain regions. The researchers proved that furin enhances *a*-secretase activity via the cleavage of ADAM10 and TACE to change to the mature forms and promotes sAPPa secretion to suppress Aβ generation.

In addition, nuclear factor erythroid 2-related factor 2 (NRF2) as a critical regulator related to ferroptosis play an important role in the development of AD. NRF2 is a transcription factor that can regulate a considerable number of genes, including genes related to iron and glutathione metabolism, which are associated with ferroptosis. NRF2 is activated to resist oxidative stress induced by excessive iron to protect tissues and cells from ferroptosis (Lim et al., 2019). The reduced levels of Nrf2 mRNA in the brains of AD patients also indicate that ferroptosis participates in the pathological process of AD. Recently reported that NRF2 as a negative regulator of BACE1, represses the expression of BACE1 by binding to antioxidant response elements (AREs) (Figure 1B). In AD patients and animal models, the mRNA levels of Nrf2 were significantly reduced, and Nrf2 deficiency accelerated BACE1 expression and Aβ production (Bahn et al., 2019). Drugs, such as spinosin, can suppress BACE1 expression by promoting Nrf2 expression and the activation of the Nrf2/HO-1 pathway, thereby inhibiting Aβ_1-42_ production (Zhang et al., 2020). In addition, iron also directly binds to the His6, His13 and His14 amino acid residues of Aβ, thereby enhancing the neurotoxicity of Aβ (Azimi and Rauk, 2012).

### Iron overload-induced tau hyperphosphorylation and NFT formation

Emerging evidence has demonstrated that there is a close connection between the abnormal tau pathology and the accumulation of iron contents in the brains of individuals with neurodegenerative disorders (Rao and Adlard, 2018). Dysregulation of brain iron homeostasis is closely related to tau hyperphosphorylation and the NFTs formation. Studies have shown that in the cortex and hippocampus of AD, NFTs accumulate with increasing iron levels in the brain regions (Spotorno et al., 2020). Tau hyperphosphorylation is a critical step for NFT formation, which is one of the important factors correlated with AD pathology (Duce et al., 2010). Tau hyperphosphorylation is regulated by the aberrant activation of tau kinases such as glycogen synthase kinase-3β (GSK3β), cyclin-dependent protein kinase-5 (CDK5) and mitogen-activated protein kinases (MAPKs). Increased cellular iron levels promote the activity of multiple kinases, such as GSK3β, CDK5, MAPK and protein phosphatase 2A, which promote tau hyperphosphorylation (Hall et al., 2011) (Figure 1C). However, intranasal DFO treatment can inhibit iron-induced tau phosphorylation via the CDK5 and GSK-3 pathways in APP/PS1 double transgenic mice (Guo et al., 2013).

In addition, the oxidation states of iron are closely related to the aggregation states of tau hyperphosphorylation. Fe^3+^ can promote the aggregation of hyperphosphorylated tau by binding to His residues of Tau (Figure 1C). However, Fe^2+^ may mediate reversible conformational changes of aggregation by binding to Thr residues of tau. Iron may function as a cofactor for tau aggregation, which causes aggregation of hyperphosphorylated tau via an iron-binding motif in the tau protein (Rao and Adlard, 2018).

Conversely, tau dysregulation also induced iron dyshomeostasis. Studies have shown that knockdown of Tau induce the accumulation of iron in the cortex, hippocampus and substantia nigra of Tau-KO mice, leading to age-dependent neurodegeneration (Lei et al., 2012). This is because iron export is mediated mainly through the ferroportin (Fpn) interaction with APP in neurons. The dysfunction of tau leads to impaired transport of APP to the membrane, which in turn affects the interaction of APP with Fpn to repress the export of iron from the cell, resulting in iron retention (Lei et al., 2012).

### Dysfunction of system Xc-induce excitotoxic neuronal degeneration in AD

The cystine/glutamate antiporter system (system Xc-), composed of the light chain SLC7A11 and the heavy chain SLC3A2, is an important glutamate transporter in the central nervous system (CNS) for the cellular uptake of cystine in exchange for intracellular glutamate. It mainly functions to mediate cellular cystine uptake for the synthesis of GSH, which is essential for cellular protection from oxidative stress. Glutamate is an important excitatory transmitter in the CNS and is critical for maintaining normal brain function and CNS development. However, glutamate levels can affect the function of system Xc-. High extracellular concentrations of glutamate disrupt system Xc- and thus induce ferroptosis.

The antioxidant properties of system Xc-are mainly achieved by increasing the synthesis of GSH. Cysteine is an important precursor in the GSH synthesis process and functions as a rate-limiting factor in the synthesis process. Studies have shown that cysteine in plasma and cerebrospinal fluid is present at a very low concentration, which is easily oxidized, and the ability to synthesize cysteine in cells is limited. Therefore, the maintenance of intracellular cysteine is mainly through the transport of cystine into the cell, where it is reduced to cysteine immediately (Tu et al., 2021). Cystine transported into astrocytes or neurons relies on system Xc-, which drives the cysteine/cystine redox cycle and protects cells from oxidative stress (Banjac et al., 2008; Lewerenz et al., 2012). However, the transport of cystine into the cell through system Xc-is accompanied by the release of a large amount of glutamate, which activates the NMDA receptors of neurons to affect the transmission of excitatory messages. Dysfunction of system Xc-not only leads to oxidative damage but also causes the release of glutamate and an increase in the extracellular glutamate concentration, resulting in neuronal glutamate excitotoxicity. In particular, the pharmacological inhibition of system Xc-by certain small molecule compounds or drugs (for example, erastin, sulfasalazine, and sorafenib) can trigger lipid peroxidation and ferroptosis.

**FIGURE 2:**
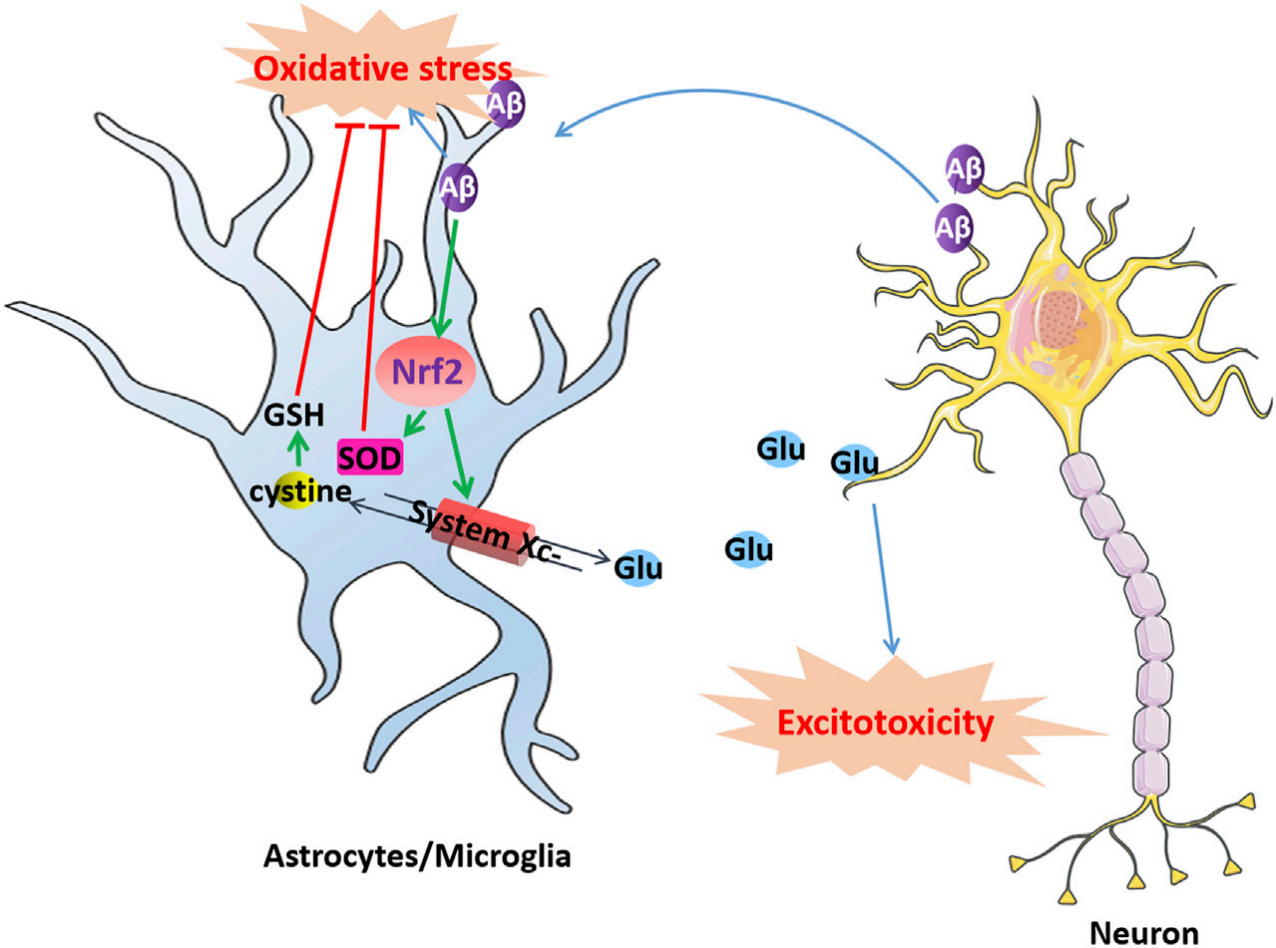
Dysfunction of system Xc-induce excitotoxic neuronal degeneration in AD. Aβ produced by neurons aggregates extracellularly to form toxic Aβ aggregates causes oxidative stress in microglia or astrocytes, which activates Nrf2 and produces SOD to resist oxidative stress. The activation of Nrf2 also can upregulate system Xc-to resist oxidative stress induced by Aβ while simultaneously releasing glutamate. However, excessively released glutamate can cause excitotoxicity in neurons, leading to cell death.

System Xc-in microglia or astrocytes might be involved in Aβ neurotoxicity (Figure 2). Aβ produced by neurons aggregates extracellularly to form toxic Aβ aggregates, which diffuse and exert their toxic effects on the surrounding cells, contributing to oxidative stress and neuroinflammation in AD. Microglia or astrocytes import l-cystine into the cell through system Xc-to synthesize GSH and resist oxidative stress induced by Aβ while simultaneously releasing glutamate. However, microglia and astrocytes in turn enhance the toxicity of Aβ to neurons by releasing excessive glutamate through system Xc-to provoke excitotoxicity. For example, D'Ezio et al. revealed that Aβ_25-35_ induced neurotoxicity by increased glutamate release was due to the increased expression of system Xc-in astrocytes. Aβ_25-35_ triggers an antioxidant response in astrocytes by inducing the activation of the Nrf2/ARE pathway and the upregulation of System Xc-. However, the upregulation of system Xc-can increase extracellular glutamate release and potentially cause excitotoxicity (D'Ezio et al., 2021). Blocking system Xc-not only protected microglia from the cytotoxicity of Aβ but also protected neurons from the excitotoxicity caused by the release of glutamate from microglia (Qin et al., 2006).

### GSH/GPX4 pathway in AD

Emerging evidence has identified GPX4 as one of the key regulators of ferroptosis in nervous cells in the brain. GPX4 is a selenoprotein glutathione peroxidase that has been proven to repair lipid hydroperoxides in membranes. Lipid peroxidation is one feature of ferroptosis-induced cell death. GPX4 functions as a repressor of 12/15-lipoxygenase-derived peroxidation, which can detoxify hydroperoxides in membrane lipids directly, thereby reducing damage to membrane function and preventing the generation of lipid peroxidation-derived reactive products such as 4-hydroxynonenal (4-HNE). GPX4 can reduce direct toxic lipid peroxide (PLOOH) to nontoxic lipid alcohols (PL-OH) using GSH as a cofactor (Imai et al., 2017). GPX4 was reported to be a regulator of ferroptosis by RSL3 and erastin, since RSL3 and erastin decrease the activity of GPX4 by directly binding to GPX4 and indirectly losing glutathione. GPX4 overexpression suppresses RSL3-induced ferroptosis, whereas GPX4 knockdown enhances the sensitivity to RSL3-induced ferroptosis, suggesting that the inhibition of GPX4 activity is a major contributor to ferroptosis.

GPX4 was identified to have reduced expression in AD. Suppression of GPX4 is one of the critical factors for ferroptosis-related AD pathogenesis. Knockout of GPX4 in the brain causes a series of features associated with AD pathology, such as cognitive impairment and hippocampal neurodegeneration in Gpx4BIKO mice (Hambright et al., 2017). Moreover, classical features closely related to ferroptosis, such as elevated lipid peroxidation, were also observed in these brain regions, and treatment with ferroptosis inhibitors improved cognitive impairment and neurodegeneration in these mice.

In addition, studies have shown that GPX4 deletion upregulates β-secretase activity and leads to elevated Aβ production. Chen et al. revealed that Gpx4^+/−^mice had higher levels of β-secretase activity elevated by increased lipid peroxidation, which resulted in the increased expression of BACE1 at the protein level (Chen et al., 2008). As previous studies have revealed, Aβ plaques and lipid peroxidation products were colocalized in the brains of AD patients. The high level of BACE1 protein led to increased amyloid plaque burdens and increased Aβ_1–40_ and Aβ_1–42_ levels in middle-aged Gpx4^+/−^mice. Therefore, Aβ peptides led to lipid peroxidation in AD. For example, lipid oxidation enzymes that catalyze the deoxygenation of PUFAs, such as lipoxygenase (LOX), cyclooxygenases (COXs), and cytochrome p450 (CYPs), have changed in AD pathology (Wu et al., 2018). In turn, lipid peroxidation products increase APP processing and Aβ production through the upregulation of BACE1.

### Dysfunction of NCOA4-mediated ferritinophagy induced ferroptosis in AD

Ferritinophagy is mediated by NCOA4, which releases free iron by the autophagic degradation of ferritin (Santana-Codina et al., 2021). NCOA4, as a selective autophagy receptor, has been identified to colocalize with endogenous LC3B and intracellular iron storage ferritin complexes. Ferritinophagy plays an important role in the physiological and pathological processes of cell growth, proliferation, differentiation, apoptosis and carcinogenesis. Some evidence has demonstrated that ferritinophagy-mediated ferroptosis is one of the critical mechanisms contributing to neurodegenerative diseases, such as AD (Quiles Del Rey and Mancias, 2019). Ferritinophagy is regulated by NCOA4, an iron-dependent protein, to maintain intracellular iron balance and exert the normal physiological functions of iron. However, excessive activation of ferritinophagy causes ferroptosis cell death. Ferroptosis can be initiated by ferritinophagy through promoting iron and ROS accumulation.

**FIGURE 3:**
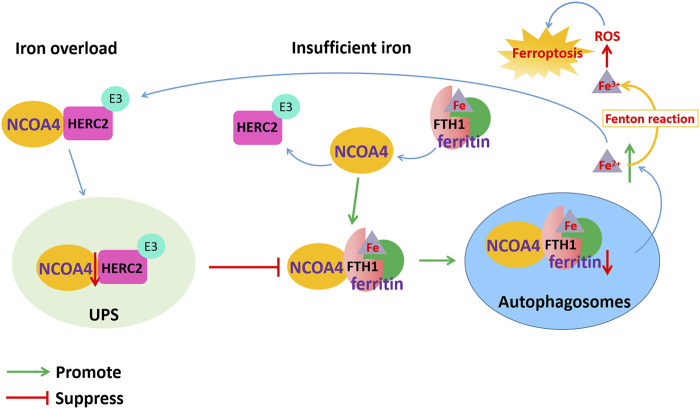
Dysfunction of NCOA4-mediated ferritinophagy induced ferroptosis in AD. NCOA4 can release free iron by the autophagic degradation of ferritin. When cellular iron is overloaded, the binding of NCOA4 and HERC2 is enhanced. HERC2 mediates the degradation of NCOA4 in an ubiquitin-dependent manner, thereby suppressing the binding of NCOA4 and ferritin and inhibiting the release of iron and ferritin degradation by ferritinophagy. When cellular iron is insufficient, ferritin-containing iron combines with NCOA4 to form a complex through FTH1 subunit of ferritin, which mediates iron release from ferritin storage through the ferritinophagy pathway. However, the excessive Fe^2+^ content in cells transforms Fe^2+^ into Fe^3+^ through the Fenton reaction to generate ROS, which causes lipid peroxidation and oxidative stress and ultimately leads to ferroptosis.

Ferritin is an iron storage protein composed of 24 subunits of two types, ferritin light chain (FTL) and heavy chain (FTH), which play an important role in maintaining iron homeostasis. When cellular iron is insufficient, ferritin-containing iron combines with NCOA4 to form a complex, which mediates iron release from ferritin storage through the ferritinophagy pathway (Santana-Codina et al., 2021). NCOA4 selectively binds to the FTH1 subunit of ferritin, and the conserved surface arginine (R23) on FTH1 is an important region that interacts with the C-terminal domain of NCOA4. When cellular iron is overloaded, the binding of NCOA4 and HERC2 (HECT and RLD domain containing E3 ubiquitin protein ligase 2) is enhanced. HERC2 is an E3 ubiquitin ligase composed of multiple structural domains that can regulate NCOA4 levels through the ubiquitin–proteasome system. The binding region of HERC2 and NCOA4 are the same as the binding region of NCOA4 and ferritin. HERC2 mediates the degradation of NCOA4 in an ubiquitin-dependent manner through the CUL7 homology domain of HERC2 and the C-terminal domain of NCOA4, thereby suppressing the binding of NCOA4 and ferritin and inhibiting the release of iron and ferritin degradation by ferritinophagy. When the available cellular iron decreases, the binding of NCOA4 to HERC2 is weakened, and NCOA4 binds to ferritin and is transported to autophagosomes, causing ferritinophagy to degrade ferritin and release free iron Fe^2+^. However, the excessive Fe^2+^ content in cells transforms Fe^2+^ into Fe^3+^ through the Fenton reaction to generate ROS, which causes lipid peroxidation and oxidative stress and ultimately leads to ferroptosis (Figure 3).

In addition, ferritinophagy dysfunction might affect the level of iron metabolism-related proteins, leading to excessive iron storage in the cells and causing ferroptosis (Santana-Codina et al., 2021). Iron is transported throughout the circulatory system by binding to Tf. Through the binding of the iron-Tf complex to the TfR, iron is then transported from serum to cells. When the depletion of NCOA4 leads to impaired ferritinophagy, the availability of cellular iron is reduced, whereas IRP2 activity is induced to bind to the IREs in the 5′-UTR of ferritin mRNA and the 3′-UTR of TfR mRNA. This inhibits the translation of ferritin mRNA yet stabilizes the TfR mRNA to promote iron transport into cells and Fe^2+^ accumulation, finally inducing ferroptosis.

## Major pathways regulating ferroptosis in AD

### Modulating NRF2 to regulate ferroptosis

Nrf2 is a master transcription factor that is conducive to improving the state of oxidative stress in the body, promoting cell survival and maintaining cellular redox homeostasis. The expression of NRF2 decreases with age, which indicates that it may play a role in the development of AD by promoting sensitivity to ferroptotic stress. In addition, the levels of many NRF2-regulated proteins such as GSH biosynthesis enzymes, GPX4, NQO1 and iron metabolism-related proteins that are linked to ferroptosis have been shown to be altered in AD (Hambright et al., 2017; Lane et al., 2021).

### Keap1/Nrf2/ARE pathway

**FIGURE 4:**
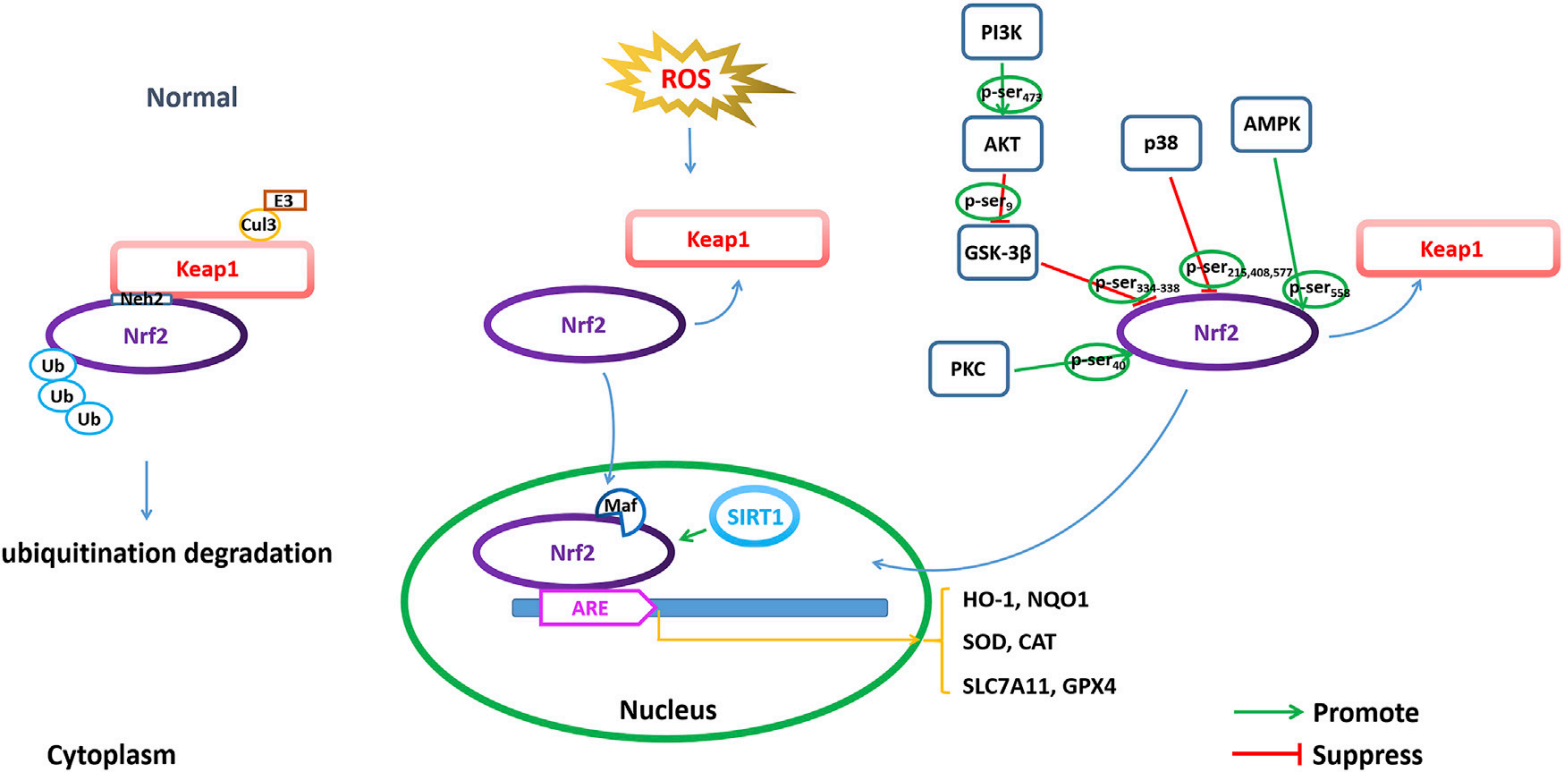
Modulating NRF2 to regulate ferroptosis. Regulation of ferroptosis through transcriptional and posttranscriptional regulation of Nrf2 is important for AD pathophysiology, including Nrf2 expression, Nrf2 phosphorylation and acetylation/deacetylation.

Nrf2 belongs to the basic leucine zipper (bZIP) transcription factor family and retains a cap-n-collar (CNC) structure. The N-terminal region of Nrf2 has a Neh2 domain and contains DLG and ETGE motifs. Under normal physiological conditions, Nrf2 interacts with Keap1 and is anchored in the cytoplasm (Bellezza et al., 2018) (Figure 4).

Keap1 is a major intracellular regulator of Nrf2, which forms a homodimer to bind Cullin3 (Cul3), an adaptor to the Cul3-type E3 ubiquitin ligase complex, resulting in ubiquitination. Under normal physiological conditions, the Neh2 domain of Nrf2 binds to the DGR region of Keap1 to be anchored in the cytoplasm. Keap1 acts as a substrate for the Cul3-dependent E3 ubiquitin ligase complex (Cul3/Rbx1 E3 ubiquitin), which can promote the ubiquitination of Nrf2 and its rapid degradation by the proteasome to maintain Nrf2 levels. Under oxidative stress conditions, such as excessive ROS production, Nrf2 disconnects from Keap1 and migrates to the nucleus, where it can dimerize with small Maf family members and bind to ARE (Bellezza et al., 2018).

The ARE is defined as a cis-acting DNA enhancer motif and is positioned in the promoter of antioxidant genes. Binding of Nrf2 to ARE regulates the transcriptional activation of many important endogenous antioxidants (such as superoxide dismutase (SOD) and enhanced catalase (CAT)) and phase II detoxifying enzymes (such as NAD(P)H quinone oxidoreductase 1 (NQO1), heme oxygenase-1 (HO-1), glutathione S-transferase A1 (GSTA1), glutamate-cysteine ligase modifier subunit (GCLM) and catalytic subunit (GCLC)) to remove harmful molecules such as ROS (Buendia et al., 2016). HO-1 is one of the detoxifying enzymes in phase II, and the downstream signaling axis induced by it has a protective effect against oxidative stress in multiple organs. In addition, the expression of SLC7A11 and GPX4 is also transcriptionally regulated by Nrf2 (Yuan et al., 2021).

### Nrf2 phosphorylation in regulating ferroptosis

The phosphorylation of Nrf2 is one of the modes by which Nrf2 nuclear accumulation and nuclear exclusion and degradation are regulated. Nrf2 comprises several sites for phosphorylation, which can be phosphorylated by many kinases (Figure 4). GSK-3β is a serine/threonine kinase that is hyperactive in the brains of AD patients. GSK-3β exerts a negative regulatory effect on Nrf2 activity by phosphorylation (Culbreth and Aschner, 2018). GSK-3β phosphorylates the Neh6 region of Nrf2 at serine residues 334–338, which is recognized by β-TrCP E3 ligase, resulting in ubiquitination and proteasomal degradation of Nrf2 (Rada et al., 2011). Activation of the PI3K/Akt pathway can indirectly regulate Nrf2 through the negative regulation of GSK-3β, while inactive GSK-3β increases Nrf2 stability and Nrf2-related gene expression. PI3K phosphorylates Akt at Ser473 to activate Akt. The phosphorylated Akt further phosphorylates GSK-3β at Ser9 and negatively regulates GSK-3β (inactivating GSK-3β), thereby suppressing GSK-3β from phosphorylating Nrf2 at Ser334-338 and promoting the activation of Nrf2 by translocation into the nucleus to initiate transcriptional activation of antioxidant enzymes (Bian et al., 2021). In addition, protein kinase C (PKC) is a serine/threonine kinase that can phosphorylate Nrf2 at Ser40 in the Neh2 domain, dissociate Nrf2 from Keap1, and promote the nuclear translocation of Nrf2, thus transcriptionally activating HO-1 expression (Huang et al., 2002; Fao et al., 2019). AMP-activated protein kinase (AMPK) is a heterotrimeric serine/threonine enzyme. AMPK can directly phosphorylate Nrf2 at Ser558 (Ser550 in mouse), which together with AMPK-mediated GSK3β inactivation promotes nuclear accumulation of Nrf2 for ARE-driven gene transactivation (Joo et al., 2016). For example, studies have shown that artether treatment can increase the expression of p-MAPK and the phosphorylation of GSK3β at the Ser9 site in both Aβ_1-42_-treated PC12 neuronal cultures and in the brain cortex of 3xTg-AD mice and upregulate the expression of Nrf2 and HO-1, thereby triggering the activation of antioxidant and anti-inflammatory genes (Li et al., 2019). Casein kinase II (CK2) is a protein involved in replication, gene transcription and transduction that can phosphorylate Nrf2 for nuclear accumulation and the activation of Nrf2 (Pi et al., 2007; Apopa et al., 2008). p38 MAPK is a class of evolutionarily conserved serine/threonine MAPKs involved in the regulation of oxidative stress. p38 MAPK can phosphorylate Nrf2 at three serine residues (Ser215, Ser408 and Ser577), which promotes decreased Nrf2 nuclear accumulation (Sun et al., 2009). Studies have shown that an increased amyloid β load in AD leads to increased levels of p38 MAPK, which are accompanied by a decrease in total Nrf2 expression as well as a decrease in accumulated Nrf2 in the nucleus. Therefore, the levels of antioxidant enzymes, such as HO-1 and NQO1, which are regulated by Nrf2, were reduced (Zhang et al., 2021a).

### SIRT1/Nrf2 pathway in ferroptosis

The balance of histone acetylation and deacetylation plays a key role in the regulation of gene expression. Lysine residues on the N-terminal of histone proteins acetylation induced by histone acetyl transferases (HATs) is associated with gene transcription, while histone deacetylation induced by histone deacetylase (HDAC) activity is associated with gene silencing (Khan et al., 2021). Acetylation or deacetylation of NRF2 by HATs and HDAC significantly changes its nuclear retention in neurons (Chang and Guarente, 2014).

Silent information regulator 1 (Sirtuin 1, SIRT1) is a class III deacetylase that is nicotinamide adenine dinucleotide (NAD+)-dependent and predominantly localized in the nucleus. SIRT1 can achieve epigenetic regulation by deacetylating lysine residues in histones. Recent studies have shown that the SIRT1/Nrf2 pathway is involved in neuroprotective effects. SIRT1-induced deacetylation promotes the nucleocytoplasmic localization and transcriptional activity of Nrf2, thereby increasing antioxidant capacity (Figure 4). A study showed that a low concentration of Aβ_25-35_ could increase the activity of SOD and the expression of SIRT1 and Nrf2 in the primary neurons and cortex of mice, indicating that a low concentration of Aβ_25-35_ could initiate antioxidant capacity by activating the SIRT1/Nrf2 pathway to induce autophagy (Zhu et al., 2021). Meanwhile, studies have also shown that vitamin D reduces oxidative stress by upregulating SIRT1, NRF-2 and HO-1 and downregulating NF-κB (Ali et al., 2021). These results indicate that increasing SIRT1 can activate the Nrf2 pathway through deacetylation by SIRT1 and trigger transcriptional activation of a series of downstream antioxidant enzymes to resist oxidative stress.

### The p53/SLC7A11 pathway regulates ferroptosis

SLC7A11 is a functional light chain subunit of the Xc-system and together with the heavy chain subunit SLC3A2 constitutes system Xc-, which is critical for ferroptosis. SLC7A11 plays a crucial role in cystine uptake that subsequently prevents ferroptosis. p53 has been reported to downregulate the expression of SLC7A11 via transcriptional regulation and to induce the iron-dependent cell death process (Wang et al., 2020a). p53 is activated by acetylation at its lysine K101 site in humans (or K98 in mouse p53), thus promoting ferroptosis (Wang et al., 2016a; Chen et al., 2021b). A study confirmed that although ablation of p53 acetylation does not significantly affect p53 expression, it does significantly abrogate p53-mediated transcriptional regulation of targets such as SLC7A11.

In addition, p53 can be deacetylated by Sirt-1, inhibiting its function of suppressing transcriptional activation of SLC7A11 and further increasing the expression of SLC7A11, thereby inhibiting ferroptosis.

### The AMPK/mTOR pathway regulates autophagy-dependent ferroptosis

AMPK is a regulator of autophagy through the mTOR signaling pathway, which can initiate chaperone-mediated autophagy and control cellular defenses against oxidative stress. mTOR is a mammalian target of rapamycin, a member of the PI3K-related kinase (PIKK) family with serine/threonine protein kinase activity. The phosphorylation of AMPK can phosphorylate mTOR to inhibit the activity of mTOR and suppress cell autophagy initiation (Ge et al., 2022). Autophagy-dependent ferroptosis is ferroptotic cell death triggered by autophagy, characterized by lipid peroxidation and iron accumulation (Liu et al., 2020). Recently, studies confirmed that the AMPK/mTOR pathway is involved in regulating autophagy-dependent ferroptosis. Inactivation of mTOR by phosphorylation of AMPK results in significantly elevated LC3B-II accumulation and beclin1 protein expression, thereby promoting autophagy and initiating autophagy-dependent ferroptotic cell death. The function of AMPK in the mediation of ferroptosis is required for beclin1 phosphorylation, with inhibition of system Xc-activity (Song et al., 2018).

Moreover, SIRT3 promotes the induction of autophagy-dependent ferroptosis by activating the AMPK-mTOR pathway. SIRT3 knockdown decreases AMPK phosphorylation, suppresses AMPK activation, and increases mTOR activation. SIRT3 silencing also significantly decreases LC3B-II accumulation and beclin1 protein expression, thereby suppressing autophagy. Depletion of SIRT3 decreases the phosphorylation level of AMPK, increases mTOR activity, and decreases LC3B-II and Beclin1 expression, eventually inhibiting autophagy activation and blocking the induction of ferroptosis (Han et al., 2020). In addition, depletion of SIRT3 inhibits ferroptosis by increasing GPX4 levels.

## Potential ferroptosis-inhibiting drugs for the treatment of AD

### Iron chelators

The iron chelators is a component with an affinity for iron ions, which can regulate the expression of iron-dependent proteins by chelating iron, thereby slowing down the pathological process of AD. Previous studies have reported several iron chelators applied in clinical studies, such as deferoxamine, minocycline and clioquinol. Unfortunately, deferoxamine and minocycline do not result in any clinically meaningful difference in the rate of decline of cognitive and functional ability, therefore, they are limited as therapeutics for AD. However, novel iron chelators are potential drugs for the treatment of AD.

[5-(N-methyl-N-propargylaminomethyl)-8-hydroxyquinoline] (M-30) is a synthesized iron chelator whose prototype is 8-hydroxyquinoline (VK-28). M-30 has propargyl monoamine oxidase (MAO) inhibitory neuroprotective and iron-chelating moieties, and it can penetrate the blood–brain barrier, which has demonstrated pro-survival neurorescue action and promoted neuronal differentiation and neuronal outgrowth. The role of M-30 may be due to its iron chelating and inhibition of membrane lipid peroxidation properties, which can prevent ferroptosis. A study showed that M-30 markedly reduced the levels of cellular APP and Aβ generation by promoting the nonamyloid pathway (Avramovich-Tirosh et al., 2007). In addition, the therapeutic efficacy of M30 for AD was also confirmed in an *in vivo* animal model. Oral pretreatment with M30 significantly prevented the development or ameliorated the already developed cognitive deficits in the STZ-induced rat model of sporadic AD. Chronic M30 treatment inhibited STZ-induced hyperphosphorylation of tau protein and decreased the expression of insulin-degrading enzyme (IDE) in the hippocampus. IDE, as a metalloprotease for the degradation of Aβ, was found to be reduced in sporadic AD (Salkovic-Petrisic et al., 2015). Therefore, M-30, as a multifunctional iron chelator drug, exhibits a potential therapeutic effect for sporadic AD.

Deferiprone is a clinically available iron chelator that recently determined the effectiveness for AD in an *in vivo* animal model. Deferiprone was determined to inhibit cognitive impairment in a scopolamine (SCOP)-induced AD rat model via an antioxidative effect (Fawzi et al., 2020). Pretreatment with deferiprone significantly attenuated the SCOP-induced increase in hippocampal and cortical acetylcholinesterase (AChE) activity in rats and decreased Aβ and iron deposition. In addition, deferiprone significantly reduced iron levels in the brain and further reduced p-tau by downregulating GSK3β and CDK-5 in the hippocampus of mice (Rao et al., 2020). This finding indicated that intervention with deferiprone may be effective in slowing disease progression in AD.

Alpha-lipoic acid (α-LA) is a naturally occurring enzyme cofactor with neuroprotective properties in AD due to its antioxidant and iron chelator properties. Earlier studies have shown that *a*-LA has the ability to slow cognitive decline in aged mice and AD mice, although it does not reduce Aβ levels or plaque deposition (Farr et al., 2003). However, *in vitro* experiments have verified that *a*-LA can inhibit the formation of Aβ fibrils (Ono et al., 2006). Further study showed that *a*-LA markedly inhibited the hyperphosphorylation of tau partly by enhancing the expression of protein phosphatase 2 A (PP2A), which could attenuate oxidative stress and ferroptosis in P301S Tau transgenic mice with tauopathy (Zhang et al., 2018).

Based on this evidence, the therapeutic effect of iron chelators on AD may be related to their ability to capture iron from Aβ and suppress Aβ aggregation (Opazo et al., 2006). Both *in vitro* and *in vivo* studies have confirmed that iron chelators can effectively alleviate ferroptosis and neuronal death caused by iron dyshomeostasis in AD and thus may be potential drugs for the treatment of AD in the clinic.

### Lipid peroxidation inhibitors

Lipid peroxidation has been shown to be an early event in AD pathogenesis and related to ferroptosis. Dyshomeostasis of intracellular iron induces the production of ROS, driving ferroptosis in AD. The molecules that reduce lipid peroxidation can be used as potential drugs against AD due to their roles in suppressing the formation of lipid peroxides.

CMS121 is a small molecule and derivative of the flavonoid fisetin and may effectively alleviate AD by inhibiting lipid peroxidation. Currently, CMS121 alleviates cognitive dysfunction in APPswe/PS1ΔE9-transgenic mice. CMS121 (34 mg/kg/day)-treated AD mice prevent excess lipid peroxidation and reduce neuroinflammation in the brain. CMS121 treatment significantly decreased the toxic lipid peroxidation product 4HNE protein in the hippocampus compared with that in AD mice and prevented Aβ accumulation and inhibited cell death. FASN, as a key enzyme in the synthesis of lipids, is a target of CMS121. A previous study showed that FASN is increased in AD patients and promotes the production of 4HNE. CMS121 suppresses the level of FASN to protect against lipid peroxidation and neuroinflammation in neurodegeneration (Ates et al., 2020). Furthermore, CMS121 has a neuroprotective role by enhancing acetyl-CoA levels to target and inhibit acetyl-CoA carboxylase 1 (ACC1), exhibiting the effect of memory enhancement in SAMP8 mice (Currais et al., 2019).

### Nrf2 agonists function as antioxidants

As a key regulator of antioxidant pathways and ferroptosis, Nrf2 exerts a critical role in AD. Some drugs including synthetic and natural compounds can promote Nrf2-mediated transcriptional regulation of the production of antioxidant enzymes and resist neuronal damage and ferroptosis caused by oxidative stress in AD. Some of the drugs currently used as Nrf2 agonists are synthetic compounds, but most are natural compounds. Regardless of the mechanism by which the compounds exert their effects, their main function is to act as antioxidants by activating the Nrf2/Keap1/HO-1 pathway.

### Synthetic compounds

The recently reported synthetic Nrf2 agonists are dimethyl fumarate (DMF), Dl-3-n-butylphthalide (Dl-NBP) and benfotiamine (BFT), and their effectiveness in treating AD has been validated *in vitro* and *in vivo* (Table 1).

**TABLE 1:** Summary of novel ferroptosis inhibitors that have been studied in AD.

Functions	Reagents	Characteristics of compounds	Study models	Targets	Tissue source	References
Iron chelators	[5-(N-methyl-N-propargylaminomethyl)-8-hydroxyquinoline] (M-30)	Synthetic compounds	Cell model; STZ-induced AD rats model	Iron ions	Cell/hippocampal tissue	73, 74
	Deferiprone	Synthetic compounds	Scopolamine-treated AD rats model; rTg (tauP301L)4510 mouse model of tauopathy	Iron ions	Hippocampal and cortical tissue	75, 76
	α-lipoic acid	Natural compounds	SAMP8 mouse model; P301S Tau transgenic mice	Iron ions	Brain tissue	77–79
Lipid peroxidation inhibitor	CMS121	Synthetic compounds	APPswe/PS1ΔE9 mouse model; SAMP8 mouse model	FASN/ACC1	Hippocampal tissue	81, 82
Nrf2 agonists	Dimethyl fumarate (DMF)	Synthetic compounds	Streptozotocin-induced AD model mice; Aβ_1-42_-induced cell model	Nrf2	Hippocampal tissue	83–85
	DL-3-n-butylphthalide (DL-NBP)	Synthetic compounds	SAMP8 mouse model; APPswe/PS1ΔE9 mouse model; P301S Tau transgenic mice	Nrf2	Hippocampal tissue	86–88
	Benfotiamine	Synthetic compounds	APPswe/PS1ΔE9 mouse model; P301S mouse model of tauopathy	Nrf2	Brain tissue	89–91
	Genistein	Natural compounds	intrahippocampal Aβ_1-40_-injected rats; Aβ25-35 induced cell model	Nrf2	Heurons	92, 93
	Quercetin	natural compounds	Aβ_25-35_ induced cell model; STZ-induced AD rats model	Nrf2	Brain tissue	94, 95
	Eriodictyol	natural compounds	Aβ_25-35_ induced cell model; APPswe/PS1ΔE9 mouse model	Nrf2	The cortex and hippocampal tissue	99–100
	5,6,7,4′-Tetramethoxyflavanone	natural compounds	Dexamethasone-induced AD mouse model; Aβ_25-35_ induced cell model	Nrf2	Brain tissue	101, 102
	Resveratrol	natural compounds	Aβ_1-42_-induced cell model; SAMP8 mouse model; mild AD patients	Nrf2/SIRT1	Brain tissue	103–105
	Cyanidin	natural compounds	Aβ_25-35_ induced cell model	Nrf2	PC12 cells/SK-N-SH cells	106, 107
	Ginkgolide B	natural compounds	SAMP8 mouse model	Nrf2	Brain tissue	108, 109
	Tetrahydroxy stilbene glycoside (TSG)	natural compounds	APPswe/PS1ΔE9 mouse model	Nrf2	The cortex and hippocampal tissue	110
	Sulforaphane	natural compounds	APPswe cell model; Aβ_1-40_ induced rat AD model	Nrf2	Hippocampal tissue	111–113
	Oxyphylla A	natural compounds	SAMP8 mouse model	Nrf2	The cortex and hippocampal tissue	53

DMF was previously shown to be a neuroprotective drug used in clinical trials on patients with relapsing-remitting multiple sclerosis. Currently, DMF is identified as a synthetic Nrf2 stimulator that has effects on antioxidant and inflammatory pathways, which might play a neuroprotective role against Aβ-induced cytotoxicity (Campolo et al., 2018). DMF inhibits ROS overproduction and reduces Aβ deposition, alleviates memory impairment and hippocampal atrophy in AD model mice and delays the progression of AD by activating the Nrf2 pathway (Sun et al., 2022). Furthermore, DMF also mitigates tauopathy in Aβ-treated cell models. DMF pretreatment suppressed tau phosphorylation at Ser396 and Aβ_1-42_-induced Thr231 by reducing the activity of GSK-3β in cells to inhibit NFT formation (Rajput et al., 2020).

Dl-NBP is a synthetic compound based on L-3-n-butylphthalide that is isolated from seeds of *Apium graveolen*s. Dl-NBP has been identified as a potentially useful drug for the treatment of AD. Early, Dl-NBP was demonstrated to reduce the levels of soluble Aβ and Aβ oligomers in the APP/PS1 mouse brain (Wang et al., 2016b). A study showed that Dl-NBP promoted the cyclic adenosine monophosphate-response element-binding protein (CREB)–CREB-binding protein (CBP) interaction and increased CBP-mediated Nrf2 acetylation, therefore activating the Nrf2 signaling pathway, reducing ROS production and attenuating oxidative stress in the APP/PS1 mouse brain (Wang et al., 2016b). Dl-NBP was found to suppress neuroinflammation and reduce Aβ secretion in Alzheimer’s-like pathology (Wang et al., 2019). Nrf2 acts as a negative regulator of thioredoxin (TrX)-interacting protein (TXNIP) and can inhibit the expression of TXNIP at a low level, thereby inhibiting the activation of NLRP3 inflammasomes. Dl-NBP treatment inhibited TXNIP-TrX signaling by regulating Nrf2, further suppressing TXNIP, enhancing TrX activity and ameliorating neuronal apoptosis and neuroinflammation in APP/PS1 mouse brains (Wang et al., 2019). Furthermore, the utility of Dl-NBP in the treatment of AD has been studied preclinically. Applying donepezil combined with Dl-NBP in the treatment of mild to moderate AD showed that after 48 weeks of treatment with donepezil combined with NBP, cognitive decline was slower in patients with AD (Wang et al., 2021).

BFT is a synthetic liposoluble derivative of vitamin B1 (also called thiamine), which has better bioavailability than thiamine. Several studies have shown that BFT can rescue cognitive deficits and reduce Aβ burden in APP/PS1 mice and AD patients. For example, a clinical study showed that oral administration of 300 mg/day BFT for 18 months to five AD patients with mild-to-moderate dementia could improve cognitive decline, despite not eliminating brain amyloid accumulation (Pan et al., 2016). Furthermore, in the APP/PS1 mouse model, BFT improved cognitive function while suppressing glycogen synthase kinase-3 (GSK-3) activity, although it did not inhibit β-secretase (BACE) activity (Pan et al., 2010). Recently study showed that BFT treatment can reverse the reduction in SOD-2 activity as well as the decrease in PGC-1a levels by activating the Nrf2/ARE transcription pathway, further decreasing GSK-3β activity and diminishing tau phosphorylation (Tapias et al., 2018).

### Natural compounds

A variety of natural compounds have been reported to act as Nrf2 agonists and have the potential to treat AD. Most of them are flavonoids (including genistein, quercetin, eriodictyol and 5,6,7,4′-tetramethoxyflavanone), polyphenols (including resveratrol and cyanidin), terpenoids (ginkgolide B) and other types of compounds. Regardless of the type of compound, their ultimate function is to promote Nrf2 expression and activation, thereby inducing the expression of antioxidant enzymes such as HO-1, NQO-1 and SOD. The specific compounds and their mechanism are shown in Table 1.

### Flavonoids

Genistein is a natural isoflavone derived from soybean extract and has been reported to have anti-inflammatory and antioxidative activities. Genistein has been identified to potentially ameliorate learning and memory deficits in AD (Bagheri et al., 2011). Recently, it was determined that genistein exerts a neuroprotective effect on an AD cell model via Nrf2/HO-1/PI3K signaling. Genistein protects against the neurotoxicity induced by Aβ_25-35_ by upregulating PI3K p85 phosphorylation to activate Nrf2, which further enhances HO-1 expression. Furthermore, a study showed that genistein treatment reversed the Aβ_25-35_-induced decrease in alpha7 nicotinic acetylcholine receptor (α7nAChR) expression in hippocampal neurons and activated the PI3K/Akt/Nrf2/keap1 signaling cascade, indicating that genistein has therapeutic potential in the prevention and treatment of AD (Guo et al., 2021).

Quercetin is one of the more common plant-derived flavonoids with antioxidant properties. Recent studies have shown that quercetin acts as an antioxidant in AD cell models and animal models. Yu et al. determined that quercetin increased the expression of sirtuin1, Nrf2 and antioxidant enzymes, thereby acting as an antioxidant in Aβ_25-35_-induced PC12 cells (Yu et al., 2020). Quercetin also attenuated cholinergic dysfunction and cognitive impairments in STZ-induced AD-like dementia rats (Singh and Garabadu, 2021). Study showed that quercetin with 50 mg/kg treatment significantly reversed STZ-induced decreases in the levels of α7nAChRs and HO-1 in the rat brain, which improved cholinergic functions and abolished the accumulation of Aβ in the brains of rats subjected to STZ. This finding indicated that quercetin can be used as a potential therapeutic drug in the management of AD by regulating the α7nAChR/Nrf2/HO-1 signaling pathway. In addition, quercetin treatment can reduce tau aggregation and protected cells against tau neurotoxicity by mediating the expression of heat shock protein family B member 1 (HSPB1), NRF2, and tropomyosin-related kinase B (TRKB) (Chiang et al., 2021). Previous studies revealed that the levels of NRF2 can be upregulated by AKT serine/threonine kinase 1 (AKT), one of the downstream proteins activated by TRKB (Yoo et al., 2017). HSPB1 regulates the status of tau protein, which is a target of NRF2 (Sahara et al., 2007).

Eriodictyol is a natural flavonoid that is widely found in citrus fruits and some Chinese herbal medicines. Eriodictyol has been found to exert antioxidant and anti-inflammatory activity. Studies showed that eriodictyol induced the activation of the Nrf2/ARE signaling pathway, leading to the upregulation of Nrf2-dependent antioxidant capacity, thereby reducing Aβ-induced oxidative damage (Jing et al., 2015). Recently, a study by Li et al. further confirmed that eriodictyol ameliorates cognitive dysfunction in APP/PS1 mice by inhibiting ferroptosis via vitamin D receptor (VDR)-mediated Nrf2 activation (Li et al., 2022). Eriodictyol treatment obviously increased the expression of GPX4 and promoted the activation of the Nrf2/HO-1 signaling pathway in the cortex and hippocampus of APP/PS1 mice. However, the effect of eriodictyol on promoting GPX4 expression and the activation of the Nrf2/HO-1 signaling pathway were reversed by VDR knockout. The results suggest that eriodictyol inhibits ferroptosis by activating the Nrf2/HO-1 signaling pathway, thereby attenuating memory impairment in AD, and this process is mediated by VDR.

5,6,7,4′-tetramethoxyflavanone (TMF) is a flavonoid isolated from *Chromolaena odorata (L.)*, which exerts antioxidation and anti-inflammation effects. Recently, TMF has been well demonstrated to have a function against neurodegeneration. For example, Jumnongprakhon et al. found that TMF attenuated Aβ_25-35_ toxicity by activating Nrf2 signaling and upregulating phase II antioxidative enzymes, further inhibiting NOX-4 activity and partially activating Sirt-1 (Jumnongprakhon et al., 2021). Pakdeepak et al. determined that TMF reduced DEX-induced Aβ generation by decreasing the expression of BACE1 and presenilin 1 (PS1) and increasing the expression of the *a*-secretase ADAM10. TMF also decreased pTau expression by inhibiting the activation of GSK-3. In addition, TMF also improved synaptic function by increasing the expression of Syn and PSD95 while decreasing acetylcholine esterase activity (Pakdeepak et al., 2020). These results suggest that TMF might have potential as a therapeutic drug for AD.

### Polyphenols

Resveratrol, a natural polyphenol derived from grapes, has been widely reported to have diverse antioxidative effects against AD. Studies show that resveratrol treatment enhanced the nuclear translation and translation of Nrf2 in the brains of aged SAMP8 mice, and it enhanced SOD, glutathione peroxidase (GSH-Px), CAT activities and HO-1 protein levels and decreased MDA content, therefore decreased Aβ level and increased ChAT level (Kong et al., 2019). Moreover, Hui et al. determined that resveratrol activated the PI3K/Akt signaling pathway, further phosphorylating Nrf2 and promoting Nrf2 translocation into the nucleus, initiating gene transcription (Hui et al., 2018). The utility of resveratrol in AD treatment has also been validated in clinical trials. A 52-week trial of resveratrol treatment in mild-moderate AD patients significantly reduced CSF Aβ_42_ levels and neuroinflammation via the activation of SIRT1. Resveratrol treatment attenuated declines in mini-mental status examination (MMSE) scores and changes in ADL (ADCS-ADL) scores (Moussa et al., 2017).

Cyanidin is a natural polyphenolic pigment widely found in plants. Previously, studies have determined that cyanidin can suppress Aβ-induced neurotoxicity in an AD cell model (Wang et al., 2016c). A recent study showed that cyanidin inhibits the TLR4 pathway by increasing the translocation of Nrf2 into the nucleus and the activation of the Nrf2 pathway, further upregulating the production of HO-1, NQO-1, GCLC, and SOD and inhibiting the NF-κB pathway. Furthermore, cyanidin could prevent Aβ-induced oxidation and neuroinflammation by targeting the TLR4/NOX4 signaling pathway, indicated that cyanidin could be used as a promising compound to the treatment of AD (Thummayot et al., 2018).

### Terpenoids

Ginkgolide B (GB) is one of the major active components derived from *Ginkgo biloba* extract and is widely used for the treatment of neurodegenerative diseases (Wang et al., 2020b). GB attenuates free radical damage and reduces inflammation, and it has a neuroprotective role in resisting ferroptosis in animal models of AD. Shao et al. determined that GB treatment for 4 weeks could effectively alleviate cognitive impairment by mitigating oxidative stress, neuroinflammation and ferroptosis in the brains of SAMP8 mice (Shao et al., 2021). GB treatment significantly reduced the Fe^2+^ content in the brains of SAMP8 mice. Moreover, GB treatment reversed the increased expression of TFR1 and NCOA4 and upregulated the expression of Nrf2 and GPX4 in the brains of SAMP8 mice, suggesting the involvement of Nrf2/GPX4 pathway-regulated ferroptosis in the GB-related protective effects on the AD mouse model.

### Others

Tetrahydroxy stilbene glycoside (TSG) is the main active substance in *Polygonum multiflorum*. Recently, TSG has been beneficial in promoting learning and memory in AD and aged mouse models. TSG treatment enhanced the antioxidant capacity and reduced Aβ_40/42_ deposition in APP/PS1 mice by promoting the activation of GSH/GPX4/ROS and Keap1/Nrf2/ARE signaling pathways, thereby inhibiting neuronal cell death and ameliorating cognitive decline in AD mice (Gao et al., 2021).

Sulforaphane is an active component extracted from cruciferous vegetables and a pharmacological activator of Nrf2, which was identified as having neuroprotective functions. Early studies have shown that sulforaphane exerts antioxidative and anti-inflammatory effects by regulating Nrf2 expression and promoting Nrf2 nuclear translocation by decreasing the DNA methylation levels of the Nrf2 promoter in an AD cell model (Zhao et al., 2018). *In vivo*, sulforaphane treatment at 40 mg/kg ameliorated neuronal injury in the hippocampi and improved the learning and cognitive ability of AD rats. At the same time, sulforaphane significantly upregulated the contents of thioredoxin and glutathione as well as the activities of antioxidant enzymes, along with the level of Nrf2, and decreased the level of Aβ_1-42_ in the cortex, hippocampus and dentate gyrus of AD rats (Zhang et al., 2021b). Another study showed that sulforaphane promoted Nrf2 nuclear translocation and increased the levels of the antioxidases HO-1 and NQO1 to exert antioxidant defenses and protective effects on cognitive impairments (Pu et al., 2018). Both *in vivo* and *in vitro* experiments have demonstrated that sulforaphane exerts an antioxidant role in AD partially through activating the Nrf2 pathway.

Oxyphylla A is a novel phenolic acid compound extracted from *Alpinia oxyphylla*, which is known as Yi Zhi in Chinese. Oxyphylla A could decrease the expression of APP, Aβ_1-40_ and Aβ_1-42_ but increase the mRNA levels of HO-1 and NQO1 and promote Nrf2 translocation from the cytoplasm to the nucleus in N2a/APP cells. Treatment with 20 mg/kg Oxyphylla A increased the expression level of Nrf2 and its downstream targets, such as HO-1 and NQO1, in both the hippocampus and cortex of mice, as well as ameliorate cognitive deficits in old SAMP8 mice (Bian et al., 2021). Thus, Oxyphylla A might exert neuroprotective effects through the Nrf2/Keap1/HO-1 pathway.

## Conclusion and perspectives

**FIGURE 5:**
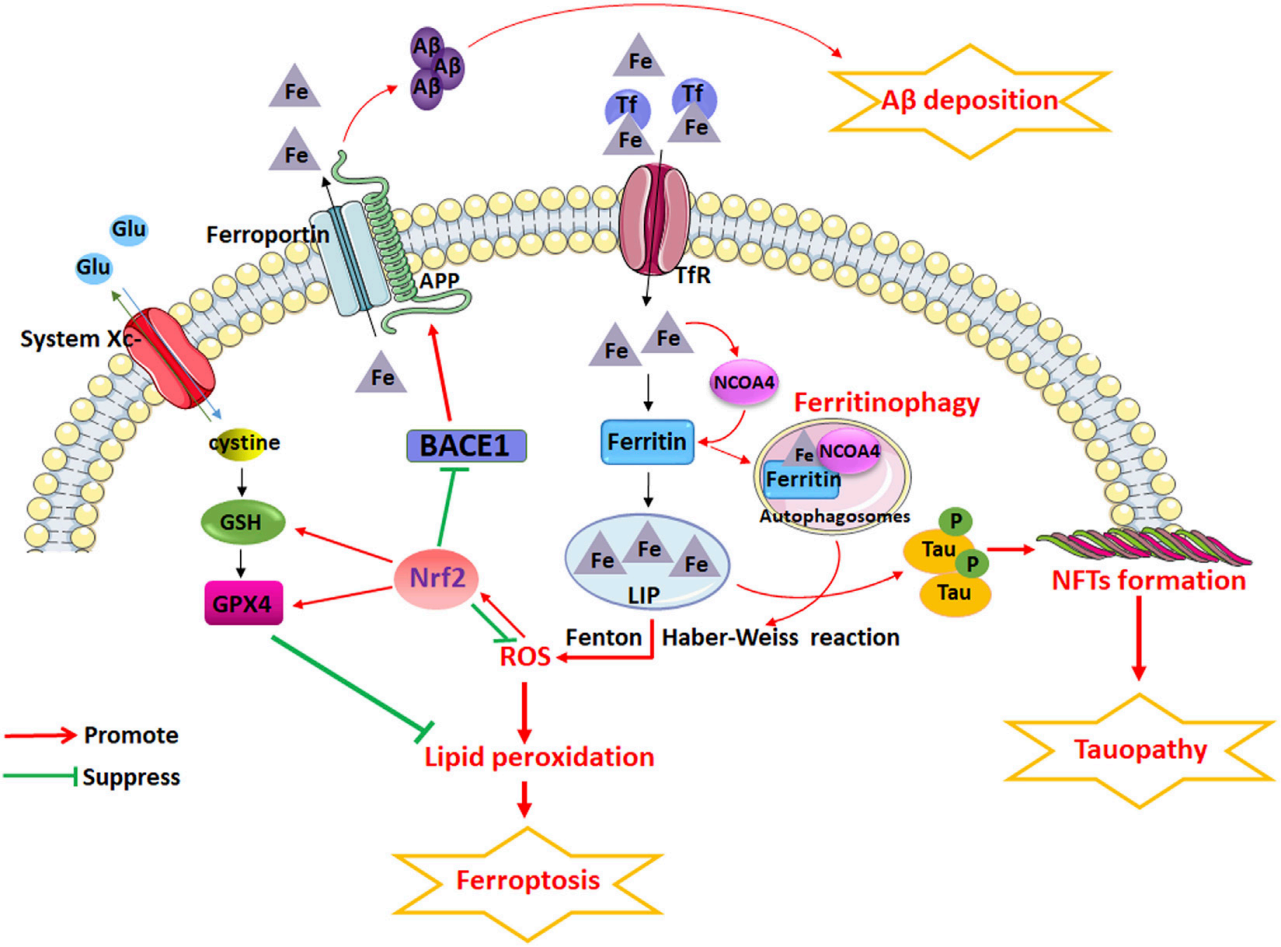
The potential role of iron dyshomeostasis and ferroptosis in AD. Iron dyshomeostasis contribute to senile plaques (SP) deposition and neurofibrillary tangles (NFTs). Iron metabolism imbalance in brain and the dysfunction of endogenous antioxidant systems including system Xc- and glutathione peroxidase (GPX) are closely related to the etiopathogenesis of AD. Dysfunction of nuclear receptor coactivator 4 (NCOA4)-mediated ferritinophagy induced ferroptosis can accelerates the pathological process of AD. However, NRF2, through regulating the expression of a considerable number of genes related to ferroptosis, including genes related to iron and glutathione metabolism, plays an important role in the development of AD.

Understanding the complex pathophysiology of AD is a priority for discovering new therapeutic targets for AD and developing novel drugs. Studies have shown that the mechanism of ferroptosis is closely related to the pathogenesis of AD, and the potential key regulatory factors that trigger ferroptosis are also plays an important role in the pathological process of AD (Figure 5). Iron dyshomeostasis contribute to SP deposition and NFTs. Iron metabolism imbalance in brain and the dysfunction of endogenous antioxidant systems including system Xc- and GPX are closely related to the etiopathogenesis of AD. Dysfunction of NCOA4-mediated ferritinophagy induced ferroptosis can accelerates the pathological process of AD. Therefore, iron dyshomeostasis and ferroptosis could be possible targets for AD therapy. The role of Nrf2 as a critical regulator of antioxidant stress has attracted much attention in recent years. NRF2, through regulating the expression of a considerable number of genes related to ferroptosis, including genes related to iron and glutathione metabolism, plays an important role in the development of AD. Future studies should explore the mechanism of iron dyshomeostasis and ferroptosis, as well as the role of Nrf2-regulated signaling pathways related to ferroptosis, in the development of AD in depth to identify new drugs that can effectively treat AD and determine their key targets.

Even though a various of molecules such as iron chelators, lipid peroxidation inhibitors and antioxidants have been found to have the potential to alleviate iron dyshomeostasis and prevent ferroptosis, more potentially effective drugs that can successfully cross the blood-brain barrier (BBB) to enter the brain and lack systemic side effects need to be identified. Discovering novel drugs and their mechanisms and applying them in clinical trials are the most important topics for future research on pharmacological treatments for AD.

In summary, the discovery of iron dyshomeostasis and ferroptosis, and the regulatory signaling pathways not only provides new insights into neuronal death in AD, but also provides an effective therapeutic target for the treatment of this disease ().

## Glossary

4-HNE: 4-hydroxynonenal
5′-UTR: 5′-untranslated region
α-LA: Alpha-lipoic acid
Aβ: beta-amyloid
ACC1: acetyl-CoA carboxylase 1
AChE: acetylcholinesterase
AD: Alzheimer’s disease
AMPK: AMP-activated protein kinase
AREs: antioxidant response elements
APP: amyloid precursor protein
BBB: blood-brain barrier
BFT: Benfotiamine
bZIP: basic leucine zipper
CAT: catalase
CBP: CREB-binding protein
CDK5: cyclin-dependent protein kinase-5
CK2: Casein kinase II
CNC: cap-n-collar
CNS: central nervous system
COXs: cyclooxygenases
CREB: cyclic adenosine monophosphate-response element-binding protein
CSF: cerebrospinal fluid
Cul3: Cullin3
CYPs: cytochrome p450
Dl-NBP: Dl-3-n-butylphthalide
DEX: dexamethasone
DFO: deferoxamine
DMF: Dimethyl fumarate
DMT1: divalent metal ion transporter 1
Fpn: ferroportin
FTH: ferritin heavy chain
FTL: ferritin light chain
GB: Ginkgolide B
GCLC: catalytic subunit
GCLM: glutamate-cysteine ligase modifier subunit
GPX4: Glutathione peroxidase 4
GSH: glutathione
GSK3β: glycogen synthase kinase-3β
GSTA1: glutathione S-transferase A1
HATs: histone acetyl transferases
HDAC: histone deacetylase
HERC2: HECT and RLD domain containing E3 ubiquitin protein ligase 2
HO-1: heme oxygenase-1
HSP1B: heat shock protein family B member 1
IDE: insulin-degrading enzyme
IREs: iron-responsive elements
IRPs: iron‐responsive‐element‐binding proteins
LIP: labile iron pool
LOX: lipoxygenase
SYN: synaptophysin
M-30: [5-(N-methyl-N-propargylaminomethyl)-8-hydroxyquinoline])
MAO: propargyl monoamine oxidase
MAPKs: mitogen-activated protein kinases
MAPT: microtubule-associated protein
MCI: mild cognitive impairment
MDA: malondialdehyde
MMSE: mini-mental status examination
nAChRs: nicotinic acetylcholine receptors
NCOA4: nuclear receptor coactivator 4
NFTs: neurofibrillary tangles
NRF2: Nuclear factor erythroid 2-related factor 2
NQO1: NAD(P)H quinone oxidoreductase 1
PIKK: PI3K-related kinase
PKC: protein kinase C
PL-OH: nontoxic lipid alcohols
PLOOH: lipid peroxide
PP2A: protein phosphatase 2A
PS1: presenilin 1
PSD95: postsynaptic density protein 95
SCOP: scopolamine
SIRT1: Silent information regulator 1 (Sirtuin 1)
SOD: superoxide dismutase
ROS: reactive oxygen species
SP: senile plaques
system Xc-: cystine/glutamate antiporter system
T2DM: type 2 diabetes mellitus
Tf: transferrin
TfR: transferrin receptor
TMF: 5,6,7,4′-tetramethoxyflavanone
TRKB: tropomyosin-related kinase B
TSG: Tetrahydroxy stilbene glycoside
TXNIP: thioredoxin (TrX)-interacting protein
VDR: vitamin D receptor

## References

[B1] Ali A. Shah S. A. Zaman N. Uddin M. N. Khan W. Ali A. (2021). Vitamin D exerts neuroprotection via SIRT1/nrf-2/NF-kB signaling pathways against D-galactose-induced memory impairment in adult mice. Neurochem. Int. 142, 104893. 10.1016/j.neuint.2020.104893 33159979

[B2] Apopa P. L. He X. Ma Q. (2008). Phosphorylation of Nrf2 in the transcription activation domain by casein kinase 2 (CK2) is critical for the nuclear translocation and transcription activation function of Nrf2 in IMR-32 neuroblastoma cells. J. Biochem. Mol. Toxicol. 22, 63–76. 10.1002/jbt.20212 18273910

[B3] Ates G. Goldberg J. Currais A. Maher P. (2020). CMS121, a fatty acid synthase inhibitor, protects against excess lipid peroxidation and inflammation and alleviates cognitive loss in a transgenic mouse model of Alzheimer's disease. Redox Biol. 36, 101648. 10.1016/j.redox.2020.101648 32863221 PMC7394765

[B4] Avramovich-Tirosh Y. Amit T. Bar-Am O. Zheng H. Fridkin M. Youdim M. B. H. (2007). Therapeutic targets and potential of the novel brain- permeable multifunctional iron chelator-monoamine oxidase inhibitor drug, M-30, for the treatment of Alzheimer's disease. J. Neurochem. 100, 490–502. 10.1111/j.1471-4159.2006.04258.x 17144902

[B5] Ayton S. Faux N. G. Bush A. I. (2015). Ferritin levels in the cerebrospinal fluid predict Alzheimer's disease outcomes and are regulated by APOE. Nat. Commun. 6, 6760. 10.1038/ncomms7760 25988319 PMC4479012

[B6] Ayton S. Portbury S. Kalinowski P. Agarwal P. Diouf I. Schneider J. A. (2021). Regional brain iron associated with deterioration in Alzheimer's disease: A large cohort study and theoretical significance. Alzheimers Dement. 17, 1244–1256. 10.1002/alz.12282 33491917 PMC9701539

[B7] Azimi S. Rauk A. (2012). The binding of Fe(II)-Heme to the amyloid beta peptide of Alzheimer's disease: QM/MM investigations. J. Chem. Theory Comput. 8, 5150–5158. 10.1021/ct300716p 26593204

[B8] Bagheri M. Joghataei M. T. Mohseni S. Roghani M. (2011). Genistein ameliorates learning and memory deficits in amyloid β(1-40) rat model of Alzheimer's disease. Neurobiol. Learn. Mem. 95, 270–276. 10.1016/j.nlm.2010.12.001 21144907

[B9] Bahn G. Park J. S. Yun U. J. Lee Y. J. Choi Y. Park J. S. (2019). NRF2/ARE pathway negatively regulates BACE1 expression and ameliorates cognitive deficits in mouse Alzheimer's models. Proc. Natl. Acad. Sci. U. S. A. 116, 12516–12523. 10.1073/pnas.1819541116 31164420 PMC6589670

[B10] Banjac A. Sato H. Seiler A. Bannai S. Weiss N. Perisic T. (2008). The cystine/cysteine cycle: A redox cycle regulating susceptibility versus resistance to cell death. Oncogene 27, 1618–1628. 10.1038/sj.onc.1210796 17828297

[B11] Belaidi A. A. Bush A. I. (2016). Iron neurochemistry in Alzheimer's disease and Parkinson's disease: Targets for therapeutics. J. Neurochem. 139 (1), 179–197. 10.1111/jnc.13425 26545340

[B12] Belaidi A. A. Gunn A. P. Wong B. X. Ayton S. Appukuttan A. T. Roberts B. R. (2018). Marked age-related changes in brain iron homeostasis in amyloid protein precursor knockout mice. Neurotherapeutics 15, 1055–1062. 10.1007/s13311-018-0656-x 30112699 PMC6277293

[B13] Bellezza I. Giambanco I. Minelli A. Donato R. (2018). Nrf2-Keap1 signaling in oxidative and reductive stress. Biochim. Biophys. Acta. Mol. Cell Res. 1865, 721–733. 10.1016/j.bbamcr.2018.02.010 29499228

[B14] Bian Y. Chen Y. Wang X. Cui G. Ung C. O. L. Lu J. H. (2021). Oxyphylla A ameliorates cognitive deficits and alleviates neuropathology via the Akt-GSK3β and Nrf2-Keap1-HO-1 pathways *in vitro* and *in vivo* murine models of Alzheimer's disease. J. Adv. Res. 34, 1–12. 10.1016/j.jare.2021.09.002 35024177 PMC8655137

[B15] Buendia I. Michalska P. Navarro E. Gameiro I. Egea J. Leon R. (2016). Nrf2-ARE pathway: An emerging target against oxidative stress and neuroinflammation in neurodegenerative diseases. Pharmacol. Ther. 157, 84–104. 10.1016/j.pharmthera.2015.11.003 26617217

[B16] Campolo M. Casili G. Lanza M. Filippone A. Paterniti I. Cuzzocrea S. (2018). Multiple mechanisms of dimethyl fumarate in amyloid beta-induced neurotoxicity in human neuronal cells. J. Cell. Mol. Med. 22, 1081–1094. 10.1111/jcmm.13358 28990726 PMC5783882

[B17] Chang H. C. Guarente L. (2014). SIRT1 and other sirtuins in metabolism. Trends Endocrinol. Metab. 25, 138–145. 10.1016/j.tem.2013.12.001 24388149 PMC3943707

[B18] Chen L. Na R. Gu M. Richardson A. Ran Q. (2008). Lipid peroxidation up-regulates BACE1 expression *in vivo*: A possible early event of amyloidogenesis in Alzheimer's disease. J. Neurochem. 107, 197–207. 10.1111/j.1471-4159.2008.05603.x 18680556 PMC2716044

[B19] Chen M. Zheng J. Liu G. Zeng C. Xu E. Zhu W. (2019). High dietary iron disrupts iron homeostasis and induces amyloid-beta and phospho-tau expression in the Hippocampus of adult wild-type and APP/PS1 transgenic mice. J. Nutr. 149, 2247–2254. 10.1093/jn/nxz168 31373375 PMC6887700

[B20] Chen X. Li J. Kang R. Klionsky D. J. Tang D. (2021). Ferroptosis: Machinery and regulation. Autophagy 17, 2054–2081. 10.1080/15548627.2020.1810918 32804006 PMC8496712

[B21] Chen W. Jiang L. Hu Y. Tang N. Liang N. Li X. F. (2021). Ferritin reduction is essential for cerebral ischemia-induced hippocampal neuronal death through p53/SLC7A11-mediated ferroptosis. Brain Res. 1752, 147216. 10.1016/j.brainres.2020.147216 33333054

[B22] Chiang N. N. Lin T. H. Teng Y. S. Sun Y. C. Chang K. H. Lin C. Y. (2021). Flavones 7, 8-DHF, quercetin, and apigenin against tau toxicity *via* activation of TRKB signaling in ΔK280 Tau_RD_-DsRed SH-SY5Y cells. Front. Aging Neurosci. 13, 758895. 10.3389/fnagi.2021.758895 34975454 PMC8714935

[B23] Crapper McLachlan D. R. Dalton A. J. Kruck T. P. Bell M. Y. Smith W. L. KaloW W. (1991). Intramuscular desferrioxamine in patients with Alzheimer's disease. Lancet 337, 1304–1308. 10.1016/0140-6736(91)92978-b 1674295

[B24] Culbreth M. Aschner M. (2018). GSK-3β, a double-edged sword in Nrf2 regulation: Implications for neurological dysfunction and disease. F1000Res. 7, 1043. 10.12688/f1000research.15239.1 30079246 PMC6053695

[B25] Currais A. Huang L. Goldberg J. Petrascheck M. Ates G. Pinto-Duarte A. (2019). Elevating acetyl-CoA levels reduces aspects of brain aging. eLife 8, e47866. 10.7554/eLife.47866 31742554 PMC6882557

[B26] D'Ezio V. Colasanti M. Persichini T. (2021). Amyloid-β 25-35 induces neurotoxicity through the up-regulation of astrocytic system X_c_<sup/>. Antioxidants 10, 1685. 10.3390/antiox10111685 34829555 PMC8615014

[B27] Dixon S. J. Lemberg K. M. Lamprecht M. R. Skouta R. Zaitsev E. M. Gleason C. E. (2012). Ferroptosis: An iron-dependent form of nonapoptotic cell death. Cell 149, 1060–1072. 10.1016/j.cell.2012.03.042 22632970 PMC3367386

[B28] Duce J. A. Tsatsanis A. Cater M. A. James S. A. Robb E. Wikhe K. (2010). Iron-export ferroxidase activity of beta-amyloid precursor protein is inhibited by zinc in Alzheimer's disease. Cell 142, 857–867. 10.1016/j.cell.2010.08.014 20817278 PMC2943017

[B29] Fao L. Mota S. I. Rego A. C. (2019). c-Src regulates Nrf2 activity through PKCδ after oxidant stimulus. Biochim. Biophys. Acta. Mol. Cell Res. 1866, 686–698. 10.1016/j.bbamcr.2019.01.011 30685263

[B30] Farr S. A. Poon H. F. Dogrukol-Ak D. Drake J. Banks W. A. Eyerman E. (2003). The antioxidants alpha-lipoic acid and N-acetylcysteine reverse memory impairment and brain oxidative stress in aged SAMP8 mice. J. Neurochem. 84, 1173–1183. 10.1046/j.1471-4159.2003.01580.x 12603840

[B31] Fawzi S. F. Menze E. T. Tadros M. G. (2020). Deferiprone ameliorates memory impairment in Scopolamine-treated rats: The impact of its iron-chelating effect on beta-amyloid disposition. Behav. Brain Res. 378, 112314. 10.1016/j.bbr.2019.112314 31644927

[B32] Gao D. Chen C. Huang R. Yang C. C. Miao B. B. Li L. (2021). Tetrahydroxy stilbene glucoside ameliorates cognitive impairments and pathology in APP/PS1 transgenic mice. Curr. Med. Sci. 41, 279–286. 10.1007/s11596-021-2344-z 33877543

[B33] Ge Y. Zhou M. Chen C. Wu X. Wang X. (2022). Role of AMPK mediated pathways in autophagy and aging. Biochimie 195, 100–113. 10.1016/j.biochi.2021.11.008 34838647

[B34] Gegotek A. Skrzydlewska E. (2019). Biological effect of protein modifications by lipid peroxidation products. Chem. Phys. Lipids 221, 46–52. 10.1016/j.chemphyslip.2019.03.011 30922835

[B35] Gerlach M. Ben-Shachar P. Riederer M. B. Youdim M. B. H. (1994). Altered brain metabolism of iron as a cause of neurodegenerative diseases? J. Neurochem. 63, 793–807. 10.1046/j.1471-4159.1994.63030793.x 7519659

[B36] Guo C. Wang P. Zhong M. L. Wang T. Huang X. S. Li J. Y. (2013). Deferoxamine inhibits iron induced hippocampal tau phosphorylation in the Alzheimer transgenic mouse brain. Neurochem. Int. 62, 165–172. 10.1016/j.neuint.2012.12.005 23262393

[B37] Guo J. Yang H. Liu Y. Liu W. Zhao R. Li H. (2021). Atomically precise silver clusterzymes protect mice from radiation damages. J. Nanobiotechnology 41, 377–393. 10.1186/s12951-021-01054-5 PMC8605545 34798888

[B38] Hall E. C. 2nd Lee S. Y. Mairuae N. Simmons Z. Connor J. R. (2011). Expression of the HFE allelic variant H63D in SH-SY5Y cells affects tau phosphorylation at serine residues. Neurobiol. Aging 32, 1409–1419. 10.1016/j.neurobiolaging.2009.08.012 19775775

[B39] Hambright W. S. Fonseca R. S. Chen L. Na R. Ran Q. (2017). Ablation of ferroptosis regulator glutathione peroxidase 4 in forebrain neurons promotes cognitive impairment and neurodegeneration. Redox Biol. 12, 8–17. 10.1016/j.redox.2017.01.021 28212525 PMC5312549

[B40] Han D. Jiang L. Gu X. Huang S. Pang J. Wu Y. (2020). SIRT3 deficiency is resistant to autophagy-dependent ferroptosis by inhibiting the AMPK/mTOR pathway and promoting GPX4 levels. J. Cell. Physiol. 235, 8839–8851. 10.1002/jcp.29727 32329068

[B41] Huang D. Liu D. Yin J. Qian T. Shrestha S. Ni H. (2017). Glutamate-glutamine and GABA in brain of normal aged and patients with cognitive impairment. Eur. Radiol. 27, 2698–2705. 10.1007/s00330-016-4669-810.1007/s00330-017-4753-8 27966041

[B42] Huang H. C. Nguyen T. Pickett C. B. (2002). Phosphorylation of Nrf2 at Ser-40 by protein kinase C regulates antioxidant response element-mediated transcription. J. Biol. Chem. 277, 42769–42774. 10.1074/jbc.M206911200 12198130

[B43] Hui Y. Chengyong T. Cheng L. Haixia H. Yuanda Z. Weihua Y. (2018). Resveratrol attenuates the cytotoxicity induced by amyloid-β1-42 in PC12 cells by upregulating heme oxygenase-1 via the PI3K/Akt/Nrf2 pathway. Neurochem. Res. 43, 297–305. 10.1007/s11064-017-2421-7 29090409

[B44] Hwang E. M. Kim S. K. Sohn J. H. Lee J. Y. Kim Y. Kim Y. S. (2006). Furin is an endogenous regulator of alpha-secretase associated APP processing. Biochem. Biophys. Res. Commun. 349, 654–659. 10.1016/j.bbrc.2006.08.077 16942750

[B45] Imai H. Matsuoka M. Kumagai T. Sakamoto T. Koumura T. (2017). Lipid peroxidation-dependent cell death regulated by GPx4 and ferroptosis. Curr. Top. Microbiol. Immunol. 403, 143–170. 10.1007/82_2016_508 28204974

[B46] Jing X. Shi H. Zhu X. Wei X. Ren M. Han M. (2015). Eriodictyol attenuates beta-amyloid 25-35 peptide-induced oxidative cell death in primary cultured neurons by activation of Nrf2. Neurochem. Res. 40, 1463–1471. 10.1007/s11064-015-1616-z 25994859

[B47] Joo M. S. Kim W. D. Lee K. Y. Kim J. H. Koo J. H. Kim S. G. (2016). AMPK facilitates nuclear accumulation of nrf2 by phosphorylating at serine 550. Mol. Cell. Biol. 36, 1931–1942. 10.1128/MCB.00118-16 27161318 PMC4936058

[B48] Jumnongprakhon P. Chokchaisiri R. Thummayot S. Suksamrarn A. Tocharus C. Tocharus J. (2021). 5, 6, 7, 4'-Tetramethoxyflavanone attenuates NADPH oxidase 1/4 and promotes sirtuin-1 to inhibit cell stress, senescence and apoptosis in Aß25-35-mediated SK-N-SH dysfunction. EXCLI J. 20, 1346–1362. 10.17179/excli2021-3841 34602929 PMC8481796

[B49] Kajarabille N. Latunde-Dada G. O. (2019). Programmed cell-death by ferroptosis: Antioxidants as mitigators. Int. J. Mol. Sci. 20, E4968. 10.3390/ijms20194968 31597407 PMC6801403

[B50] Khan H. Tiwari P. Kaur A. Singh T. G. (2021). Sirtuin acetylation and deacetylation: A complex paradigm in neurodegenerative disease. Mol. Neurobiol. 58, 3903–3917. 10.1007/s12035-021-02387-w 33877561

[B51] Kong D. Yan Y. He X. Y. Yang H. Liang B. Wang J. (2019). Effects of resveratrol on the mechanisms of antioxidants and estrogen in Alzheimer's disease. Biomed. Res. Int. 2019, 8983752. 10.1155/2019/8983752 31016201 PMC6446083

[B52] Lane D. J. R. Metselaar B. Greenough M. Bush A. I. Ayton S. J. (2021). Ferroptosis and NRF2: An emerging battlefield in the neurodegeneration of Alzheimer's disease. Essays Biochem. 65, 925–940. 10.1042/EBC20210017 34623415

[B53] Lei P. Ayton S. Finkelstein D. I. Spoerri L. Ciccotosto G. D. Wright D. K. (2012). Tau deficiency induces parkinsonism with dementia by impairing APP-mediated iron export. Nat. Med. 18, 291–295. 10.1038/nm.2613 22286308

[B54] Lewerenz J. Maher P. Methner A. (2012). Regulation of xCT expression and system x (c) (-) function in neuronal cells. Amino acids 42, 171–179. 10.1007/s00726-011-0862-x 21369940

[B55] Li S. Zhao X. Lazarovici P. Zheng W. (2019). Artemether activation of AMPK/GSK3β(ser9)/Nrf2 signaling confers neuroprotection towards β-amyloid-induced neurotoxicity in 3xTg Alzheimer's mouse model. Oxid. Med. Cell. Longev. 2019, 1862437. 10.1155/2019/1862437 31871541 PMC6907052

[B56] Li L. Li W. J. Zheng X. R. Liu Q. L. Du Q. Lai Y. J. (2022). Eriodictyol ameliorates cognitive dysfunction in APP/PS1 mice by inhibiting ferroptosis via vitamin D receptor-mediated Nrf2 activation. Mol. Med. 28, 11. 10.1186/s10020-022-00442-3 35093024 PMC8800262

[B57] Lim P. J. Duarte T. L. Arezes J. Garcia-Santos D. Hamdi A. Pasricha S. R. (2019). Nrf2 controls iron homeostasis in haemochromatosis and thalassaemia via Bmp6 and hepcidin. Nat. Metab. 1, 519–531. 10.1038/s42255-019-0063-6 31276102 PMC6609153

[B58] Liu J. Kuang F. Kroemer G. Klionsky D. J. Kang R. Tang D. (2020). Autophagy-dependent ferroptosis: Machinery and regulation. Cell Chem. Biol. 27, 420–435. 10.1016/j.chembiol.2020.02.005 32160513 PMC7166192

[B59] Lyras L. Cairns N. J. Jenner A. Jenner P. Halliwell B. (1997). An assessment of oxidative damage to proteins, lipids, and DNA in brain from patients with Alzheimer's disease. J. Neurochem. 68, 2061–2069. 10.1046/j.1471-4159.1997.68052061.x 9109533

[B60] Moussa C. Hebron M. Huang X. Ahn J. Rissman R. A. Aisen P. S. (2017). Resveratrol regulates neuro-inflammation and induces adaptive immunity in Alzheimer's disease. J. Neuroinflammation 14, 1. 10.1186/s12974-016-0779-0 28086917 PMC5234138

[B61] O'Brien R. J. Wong P. C. (2011). Amyloid precursor protein processing and Alzheimer's disease. Annu. Rev. Neurosci. 34, 185–204. 10.1146/annurev-neuro-061010-113613 21456963 PMC3174086

[B62] Ono K. Hirohata M. Yamada M. (2006). Alpha-lipoic acid exhibits anti-amyloidogenicity for beta-amyloid fibrils *in vitro* . Biochem. Biophys. Res. Commun. 341, 1046–1052. 10.1016/j.bbrc.2006.01.063 16460684

[B63] Opazo C. Luza S. Villemagne V. L. Volitakis I. Rowe C. Barnham K. J. (2006). Radioiodinated clioquinol as a biomarker for beta-amyloid: Zn complexes in Alzheimer's disease. Aging Cell 5, 69–79. 10.1111/j.1474-9726.2006.00196.x 16441845

[B64] Pakdeepak K. Chokchaisiri R. Tocharus J. Jearjaroen P. Tocharus C. Suksamrarn A. (2020). 5, 6, 7, 4'-Tetramethoxyflavanone protects against neuronal degeneration induced by dexamethasone by attenuating amyloidogenesis in mice. EXCLI J. 19, 16–32. 10.17179/excli2019-1940 32038114 PMC7003641

[B65] Pan X. Gong N. Zhao J. Yu Z. Gu F. Chen J. (2010). Powerful beneficial effects of benfotiamine on cognitive impairment and beta-amyloid deposition in amyloid precursor protein/presenilin-1 transgenic mice. Brain 133, 1342–1351. 10.1093/brain/awq069 20385653

[B66] Pan X. Chen Z. Fei G. Pan S. Bao W. Ren S. (2016). Long-term cognitive improvement after benfotiamine administration in patients with Alzheimer's disease. Neurosci. Bull. 32, 591–596. 10.1007/s12264-016-0067-0 27696179 PMC5567484

[B67] Park E. Chung S. W. (2019). ROS-mediated autophagy increases intracellular iron levels and ferroptosis by ferritin and transferrin receptor regulation. Cell Death Dis. 10, 822. 10.1038/s41419-019-2064-5 31659150 PMC6817894

[B68] Pi J. Bai Y. Reece J. M. Williams J. Liu D. Freeman M. L. (2007). Molecular mechanism of human Nrf2 activation and degradation: Role of sequential phosphorylation by protein kinase CK2. Free Radic. Biol. Med. 42, 1797–1806. 10.1016/j.freeradbiomed.2007.03.001 17512459 PMC1950666

[B69] Pu D. Zhao Y. Chen J. Sun Y. Lv A. Zhu S. (2018). Protective effects of sulforaphane on cognitive impairments and AD-like lesions in diabetic mice are associated with the upregulation of Nrf2 transcription activity. Neuroscience 381, 35–45. 10.1016/j.neuroscience.2018.04.017 29684505

[B70] Qin S. Colin C. Hinners I. Gervais A. Cheret C. Mallat M. (2006). System Xc- and apolipoprotein E expressed by microglia have opposite effects on the neurotoxicity of amyloid-beta peptide 1-40. J. Neurosci. 26, 3345–3356. 10.1523/JNEUROSCI.5186-05.2006 16554485 PMC6674113

[B71] Quiles Del Rey M. Mancias J. D. (2019). NCOA4-Mediated ferritinophagy: A potential link to neurodegeneration. Front. Neurosci. 13, 238. 10.3389/fnins.2019.00238 30930742 PMC6427834

[B72] Rada P. Rojo A. I. Chowdhry S. McMahon M. Hayes J. D. Cuadrado A. (2011). SCF/{beta}-TrCP promotes glycogen synthase kinase 3-dependent degradation of the Nrf2 transcription factor in a Keap1-independent manner. Mol. Cell. Biol. 31, 1121–1133. 10.1128/MCB.01204-10 21245377 PMC3067901

[B73] Rajput M. S. Nirmal N. P. Rathore D. Dahima R. (2020). Dimethyl fumarate mitigates tauopathy in aβ-induced neuroblastoma SH-SY5Y cells. Neurochem. Res. 45, 2641–2652. 10.1007/s11064-020-03115-x 32816241

[B74] Ramos P. Santos A. Pinto N. R. Mendes R. Magalhaes T. Almeida A. (2014). Iron levels in the human brain: A post-mortem study of anatomical region differences and age-related changes. J. Trace Elem. Med. Biol. 28, 13–17. 10.1016/j.jtemb.2013.08.001 24075790

[B75] Rao S. S. Adlard P. A. (2018). Untangling tau and iron: Exploring the interaction between iron and tau in neurodegeneration. Front. Mol. Neurosci. 11, 276. 10.3389/fnmol.2018.00276 30174587 PMC6108061

[B76] Rao S. S. Portbury S. D. Lago L. Bush A. I. Adlard P. A. (2020). The iron chelator deferiprone improves the phenotype in a mouse model of tauopathy. J. Alzheimers Dis. 77, 1783–1787. 10.3233/JAD-209009 33252085

[B77] Rogers J. T. Bush A. I. Cho H. H. Smith D. H. Thomson A. M. Friedlich A. L. (2008). Iron and the translation of the amyloid precursor protein (APP) and ferritin mRNAs: Riboregulation against neural oxidative damage in Alzheimer's disease. Biochem. Soc. Trans. 36, 1282–1287. 10.1042/BST0361282 19021541 PMC2746665

[B78] Sahara N. Maeda S. Yoshiike Y. Mizoroki T. YamaShita S. MurayaMaM. (2007). Molecular chaperone-mediated tau protein metabolism counteracts the formation of granular tau oligomers in human brain. J. Neurosci. Res. 85, 3098–3108. 10.1002/jnr.21417 17628496

[B79] Salkovic-Petrisic M. Knezovic A. Osmanovic-Barilar J. Smailovic U. Trkulja V. Riederer P. (2015). Multi-target iron-chelators improve memory loss in a rat model of sporadic Alzheimer's disease. Life Sci. 136, 108–119. 10.1016/j.lfs.2015.06.026 26159898

[B80] Santana-Codina N. Gikandi A. Mancias J. D. (2021). The role of NCOA4-mediated ferritinophagy in ferroptosis. Adv. Exp. Med. Biol. 1301, 41–57. 10.1007/978-3-030-62026-4_4 34370287

[B81] Senyilmaz D. Virtue S. Xu X. Tan C. Y. Griffin J. L. Miller A. K. (2015). Regulation of mitochondrial morphology and function by stearoylation of TFR1. Nature 525, 124–128. 10.1038/nature14601 26214738 PMC4561519

[B82] Shao L. Dong C. Geng D. He Q. Shi Y. (2021). Ginkgolide B protects against cognitive impairment in senescence-accelerated P8 mice by mitigating oxidative stress, inflammation and ferroptosis. Biochem. Biophys. Res. Commun. 572, 7–14. 10.1016/j.bbrc.2021.07.081 34332327

[B83] Singh N. K. Garabadu D. (2021). Quercetin exhibits α7nAChR/nrf2/HO-1-Mediated neuroprotection against STZ-induced mitochondrial toxicity and cognitive impairments in experimental rodents. Neurotox. Res. 39, 1859–1879. 10.1007/s12640-021-00410-5 34554409

[B84] Song X. Zhu S. Chen P. Hou W. Wen Q. Liu J. (2018). AMPK-mediated BECN1 phosphorylation promotes ferroptosis by directly blocking system Xc(-) activity. Curr. Biol. 28, 2388–2399. e2385. 10.1016/j.cub.2018.05.094 30057310 PMC6081251

[B85] Spotorno N. Acosta-Cabronero J. Stomrud E. Lampinen B. Strandberg O. T. van Westen D. (2020). Relationship between cortical iron and tau aggregation in Alzheimer's disease. Brain 143, 1341–1349. 10.1093/brain/awaa089 32330946 PMC7241946

[B86] Sun Z. Huang Z. Zhang D. D. (2009). Phosphorylation of Nrf2 at multiple sites by MAP kinases has a limited contribution in modulating the Nrf2-dependent antioxidant response. PloS one 4, e6588. 10.1371/journal.pone.0006588 19668370 PMC2719090

[B87] Sun X. Suo X. Xia X. Yu C. Dou Y. (2022). Dimethyl fumarate is a potential therapeutic option for Alzheimer's disease. J. Alzheimers Dis. 85, 443–456. 10.3233/JAD-215074 34842188

[B88] Svobodova H. Kosnac D. BalaZsiova Z. Tanila H. Miettinen P. O. SierrA A. (2019). Elevated age-related cortical iron, ferritin and amyloid plaques in APP(swe)/PS1(deltaE9) transgenic mouse model of Alzheimer's disease. Physiol. Res. 68, S445–S451. 10.33549/physiolres.934383 32118475

[B89] Tapias V. Jainuddin S. Ahuja M. Stack C. Elipenahli C. Vignisse J. (2018). Benfotiamine treatment activates the Nrf2/ARE pathway and is neuroprotective in a transgenic mouse model of tauopathy. Hum. Mol. Genet. 27, 2874–2892. 10.1093/hmg/ddy201 29860433 PMC6077804

[B90] Thummayot S. Tocharus C. Jumnongprakhon P. Suksamrarn A. Tocharus J. (2018). Cyanidin attenuates Aβ25-35-induced neuroinflammation by suppressing NF-κB activity downstream of TLR4/NOX4 in human neuroblastoma cells. Acta Pharmacol. Sin. 39, 1439–1452. 10.1038/aps.2017.203 29671417 PMC6289386

[B91] Tu H. Tang L. J. Luo X. J. Ai K. L. Peng J. (2021). Insights into the novel function of system Xc- in regulated cell death. Eur. Rev. Med. Pharmacol. Sci. 25, 1650–1662. 10.26355/eurrev_202102_24876 33629335

[B92] van Bergen J. M. Li X. Hua J. Schreiner S. J. Steininger S. C. Quevenco F. C. (2016). Colocalization of cerebral iron with amyloid beta in mild cognitive impairment. Sci. Rep. 6, 35514. 10.1038/srep35514 27748454 PMC5066274

[B93] Wang S. J. Li D. Ou Y. Jiang L. Chen Y. Zhao Y. (2016). Acetylation is crucial for p53-mediated ferroptosis and tumor suppression. Cell Rep. 17, 366–373. 10.1016/j.celrep.2016.09.022 27705786 PMC5227654

[B94] Wang C. Y. Wang Z. Y. Xie J. W. Wang T. Wang X. Xu Y. (2016). Dl-3-n-butylphthalide-induced upregulation of antioxidant defense is involved in the enhancement of cross talk between CREB and Nrf2 in an Alzheimer's disease mouse model. Neurobiol. Aging 38, 32–46. 10.1016/j.neurobiolaging.2015.10.024 26827641

[B95] Wang Y. Fu X. T. Li D. W. Wang K. Wang X. Z. Li Y. (2016). Cyanidin suppresses amyloid beta-induced neurotoxicity by inhibiting reactive oxygen species-mediated DNA damage and apoptosis in PC12 cells. Neural Regen. Res. 11, 795–800. 10.4103/1673-5374.182707 27335564 PMC4904471

[B96] Wang C. Y. Xu Y. Wang X. Guo C. Wang T. Wang Z. Y. (2019). Dl-3-n-Butylphthalide inhibits NLRP3 inflammasome and mitigates alzheimer's-like pathology via nrf2-TXNIP-TrX Axis. Antioxid. Redox Signal. 30, 1411–1431. 10.1089/ars.2017.7440 29634349

[B97] Wang T. Li X. Sun S. L. (2020). EX527, a Sirt-1 inhibitor, induces apoptosis in glioma via activating the p53 signaling pathway. Anticancer. Drugs 31, 19–26. 10.1097/CAD.0000000000000824 31490284

[B98] Wang L. Lei Q. Zhao S. Xu W. Dong W. Ran J. (2020). Ginkgolide B maintains calcium homeostasis in hypoxic hippocampal neurons by inhibiting calcium influx and intracellular calcium release. Front. Cell. Neurosci. 14, 627846. 10.3389/fncel.2020.627846 33679323 PMC7928385

[B99] Wang J. Guo X. Lu W. Liu J. Zhang H. Quan Q. (2021). Donepezil combined with DL-3-n-butylphthalide delays cognitive decline in patients with mild to moderate Alzheimer's disease: A multicenter, prospective cohort study. J. Alzheimers Dis. 80, 673–681. 10.3233/JAD-201381 33579850

[B100] Wang Y. Chen G. Shao W. (2022). Identification of ferroptosis-related genes in Alzheimer's disease based on bioinformatic analysis. Front. Neurosci. 16, 823741. 10.3389/fnins.2022.823741 35197821 PMC8858973

[B101] Ward R. J. Zucca F. A. Duyn J. H. Crichton R. R. Zecca L. (2014). The role of iron in brain ageing and neurodegenerative disorders. Lancet. Neurol. 13, 1045–1060. 10.1016/S1474-4422(14)70117-6 25231526 PMC5672917

[B102] Wichaiyo S. Yatmark P. Morales Vargas R. E. Sanvarinda P. Svasti S. Fucharoen S. (2015). Effect of iron overload on furin expression in wild-type and beta-thalassemic mice. Toxicol. Rep. 2, 415–422. 10.1016/j.toxrep.2015.01.004 28962376 PMC5598392

[B103] Wu J. R. Tuo Q. Z. Lei P. (2018). Ferroptosis, a recent defined form of critical cell death in neurological disorders. J. Mol. Neurosci. 66, 197–206. 10.1007/s12031-018-1155-6 30145632

[B104] Yoo J. M. Lee B. D. Sok D. E. Ma J. Y. Kim M. R. (2017). Neuroprotective action of N-acetyl serotonin in oxidative stress-induced apoptosis through the activation of both TrkB/CREB/BDNF pathway and Akt/Nrf2/Antioxidant enzyme in neuronal cells. Redox Biol. 11, 592–599. 10.1016/j.redox.2016.12.034 28110215 PMC5247570

[B105] Yu X. Li Y. Mu X. (2020). Effect of quercetin on PC12 Alzheimer's disease cell model induced by Aβ 25-35 and its mechanism based on sirtuin1/nrf2/HO-1 pathway. Biomed. Res. Int. 2020, 8210578. 10.1155/2020/8210578 32420373 PMC7201675

[B106] Yuan Y. Zhai Y. Chen J. Xu X. Wang H. (2021). Kaempferol ameliorates oxygen-glucose deprivation/reoxygenation-induced neuronal ferroptosis by activating nrf2/slc7a11/GPX4 Axis. Biomolecules 11, 923. 10.3390/biom11070923 34206421 PMC8301948

[B107] Zhang C. W. Tai Y. K. Chai B. H. Chew K. C. M. Ang E. T. Tsang F. (2017). Transgenic mice overexpressing the divalent metal transporter 1 exhibit iron accumulation and enhanced parkin expression in the brain. Neuromolecular Med. 19, 375–386. 10.1007/s12017-017-8451-0 28695462 PMC5570798

[B108] Zhang Y. H. Wang D. W. Xu S. F. Zhang S. Fan Y. G. Yang Y. Y. (2018). α-Lipoic acid improves abnormal behavior by mitigation of oxidative stress, inflammation, ferroptosis, and tauopathy in P301S Tau transgenic mice. Redox Biol. 14, 535–548. 10.1016/j.redox.2017.11.001 29126071 PMC5684493

[B109] Zhang X. Wang J. Gong G. Ma R. Xu F. Yan T. (2020). Spinosin inhibits aβ1-42 production and aggregation via activating Nrf2/HO-1 pathway. Biomol. Ther. 28, 259–266. 10.4062/biomolther.2019.123 PMC7216747 31791116

[B110] Zhang X. Liang S. Gao X. Huang H. Lao F. Dai X. (2021). Protective effect of chitosan oligosaccharide against hydrogen peroxide-mediated oxidative damage and cell apoptosis via activating Nrf2/ARE signaling pathway. Neurotox. Res. 39, 1708–1720. 10.1007/s12640-021-00419-w 34622385

[B111] Zhang S. Zhao J. Bai Z. Luo L. Wu F. Li B. (2021). Sulforaphane inhibits the production of Aβ partially through the activation of Nrf2-regulated oxidative stress. Food Funct. 12, 11482–11490. 10.1039/d1fo02651h 34699582

[B112] Zhao F. Zhang J. Chang N. (2018). Epigenetic modification of Nrf2 by sulforaphane increases the antioxidative and anti-inflammatory capacity in a cellular model of Alzheimer's disease. Eur. J. Pharmacol. 824, 1–10. 10.1016/j.ejphar.2018.01.046 29382536

[B113] Zhu L. Lu F. Jia X. Yan Q. Zhang X. Mu P. (2021). Amyloid-beta (25-35) regulates neuronal damage and memory loss via SIRT1/Nrf2 in the cortex of mice. J. Chem. Neuroanat. 114, 101945. 10.1016/j.jchemneu.2021.101945 33716102

